# Soft Sensor Development for Real-Time Process Monitoring of Multidimensional Fractionation in Tubular Centrifuges

**DOI:** 10.3390/nano11051114

**Published:** 2021-04-25

**Authors:** Marvin Winkler, Marco Gleiss, Hermann Nirschl

**Affiliations:** Institute of Mechanical Process Engineering and Mechanics, Karlsruhe Institute of Technology (KIT), Strasse am Forum 8, 76131 Karlsruhe, Germany; marco.gleiss@kit.edu (M.G.); hermann.nirschl@kit.edu (H.N.)

**Keywords:** solid–liquid separation, multidimensional particle features, tubular centrifuge, process monitoring, soft sensor, UV/vis, chemometrics

## Abstract

High centrifugal acceleration and throughput rates of tubular centrifuges enable the solid–liquid size separation and fractionation of nanoparticles on a bench scale. Nowadays, advantageous product properties are defined by precise specifications regarding particle size and material composition. Hence, there is a demand for innovative and efficient downstream processing of complex particle suspensions. With this type of centrifuge working in a semi-continuous mode, an online observation of the separation quality is needed for optimization purposes. To analyze the composition of fines downstream of the centrifuge, a UV/vis soft sensor is developed to monitor the sorting of polymer and metal oxide nanoparticles by their size and density. By spectroscopic multi-component analysis, a measured UV/vis signal is translated into a model based prediction of the relative solids volume fraction of the fines. High signal stability and an adaptive but mandatory calibration routine enable the presented setup to accurately predict the product’s composition at variable operating conditions. It is outlined how this software-based UV/vis sensor can be utilized effectively for challenging real-time process analytics in multi-component suspension processing. The setup provides insight into the underlying process dynamics and assists in optimizing the outcome of separation tasks on the nanoscale.

## 1. Introduction

Separation and sorting of submicron- and nanosized particulates have received a lot of research attention in recent years. Elaborate synthesis [1,2,3] and mechanical formulation strategies [4,5] in the liquid phase are able to produce well defined nanoparticles (NPs) applicable in, for example, the field of biomedicine [6,7,8,9], catalysis [10,11] and electronics [12,13,14]. Other strategies even pursue their usage as building blocks for novel materials [15,16,17].

Their advantageous properties are often tied to a specific size, shape or composition with low tolerance concerning polydispersity [18,19,20,21,22,23,24,25]. Hence, manufacturing processes commonly require subsequent purification steps like classification (sorting by size) or fractionation (sorting by other parameters than size) either to adjust a key property distribution or to isolate a specific fraction from by-products or pollutants. One of the most important unit operations in colloidal science is centrifugation of NPs in a lower-density liquid. High centrifugal forces accelerate the particles movement along a radial axis, which is, in turn, dependent on the NP material, size and shape. Remarkable separation qualities have been recorded in the literature as a result of using advanced methods such as density gradient centrifugation [26,27,28,29]. Disadvantages of these single batch procedures, however, are limitations in large scale application as well as the fact that they sacrifice high throughput and cost efficiency for product purity in small batch sizes [30,31,32].

The design of tubular centrifuges combines a semi-continuous operation with high rotor speeds enabling purification and screening processes of submicron and nanosized particles on a bench scale [33,34,35,36,37,38]. Despite an enhanced suspension processing rate, continuous sediment build-up inside the rotor leads to a decrease in separation efficiency and product quality over time. Hence, purposeful process monitoring and control of the centrifuges operating parameters is required. The study carried out by Konrath et al. [39] revealed that a turbidity measurement is sufficient for the univariate concentration monitoring of a single-component in nanoparticle classification resulting in the sought-out consistency in cut size. The question arises whether this principle can be extended to more complex fractionation tasks.

In general, separation monitoring in semi-continuous processes seeks to evaluate the product properties and quality efficiently. Consequently, the mass of individual species and their particle size distribution (PSD) are simultaneously considered as target variables, helping to identify and guarantee a certain process outcome [40]. In a best case scenario, this valuable information is derived in situ from process data during particle processing as opposed to a costly and delayed laboratory analysis. Data-driven soft sensors offer a sophisticated approach to target quantity monitoring using historical process data and regression algorithms. Their real-time processing of plant data (temperature, pressure, turbidity, etc.) allows them to be tailored reliably towards a given process condition [41,42]. However, regarding the above-mentioned analysis principles based on light scattering in a turbidity measurement, it is difficult to assign process data to single constituents sharing the same properties on a chemical level or the nanoscale. In other words, implementation of a multivariate approach is needed to ensure constant separation quality. Ample evidence exists to support the hypothesis that multi-wavelength UV/vis spectroscopy is suitable for particle concentration monitoring in solid–liquid dispersion both ex situ [43,44,45,46] and in situ [47,48,49,50,51]. Previous research in the field of semi-continuous separation did not emphasize the importance of an elaborate technique to monitor the suspension composition but rather focused on the total amount of solids (TAS) in the apparatus downstream [39,52].

In light of this, the aim of this study is twofold. The first is to address the issue of continuous composition monitoring in tubular centrifuges by establishing a working UV/vis soft sensor located downstream. Here, wet dispersions of polydisperse products and their mixtures function as model systems. The second is to provide detailed information on the inferential model structure combining spectroscopic data preparation, feature selection, training and validation procedures in order to perform accurate predictions on suspension composition based on multi-wavelength extinction data measured in real-time during classification or fractionation.

This paper is structured into several parts. Section 2 first outlines the theoretical background of both separation in tubular centrifuges and spectroscopic multi-component analysis. Afterward, Section 3 provides detailed information on the process layout, the model particle systems and the underlying software structure of the sensor. In Section 4, calibration data set generation and a quality assessment of the incorporated regression model is presented. Finally, the prediction algorithm is applied in single-component classification and multi-component fractionation monitoring. The study shows that with a carefully supervised soft sensor design, continuous and quantitative analysis of the product’s material composition in the centrifuge overflow is feasible.

## 2. Theory

### 2.1. Fractionation of Nanoparticles in Tubular Centrifuges

**Figure 1:**
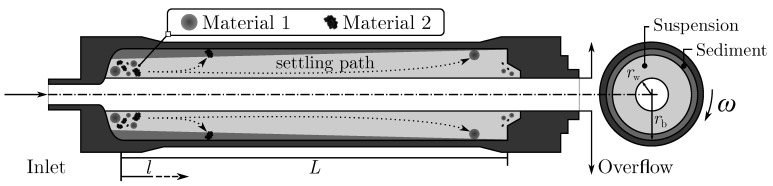
Schematic view of a tubular centrifuge rotor illustrating the separation zone with its length *L*, exemplary settling paths of two suspended materials (**left**) and an axial cross section (**right**).

A smooth, dispersed solid matter in creeping motion inside a tubular centrifuge experiences mass and frictional forces as it settles along the radial and axial coordinates inside a fluid reservoir. The suspension is fed into the centrifuge rotor with a constant volumetric flow rate ${\dot{V}}_{f}$. By assuming plug flow, a particles residence time

(1) $$t_{\mathrm{res}}=\frac{V}{{\dot{V}}_{f}}=\frac{\pi \cdot \left(r_{b}^{2}-r_{w}^{2}\right)\cdot l}{{\dot{V}}_{f}}$$

traveling a distance *l* inside the centrifuge is altered by the liquid throughput ${\dot{V}}_{f}$ and the available surface area $A=\pi \cdot \left(r_{b}^{2}-r_{w}^{2}\right)$ of the formed liquid pool. A graphical representation of this cross section and two exemplary settling paths are shown on the left hand side in Figure 1. The tubes length *L*, wall $r_{b}$ and weir radius $r_{w}$ constitute the geometric boundaries during separation.

A second process parameter denoted as the centrifugal number $C=\frac{\omega^{2}\cdot r}{g}$ indicates the amplified gravitational field strength and is calculated with the angular velocity of the rotor ω, a reference radius *r* and the gravitational constant *g*. In the following, the wall radius $r_{b}$ is used to calculate *C*. For uncharged, spherical colloids in an infinitely diluted suspension with no solid–liquid or solid–solid interactions, the state of force equilibrium between drag, buoyancy, and centrifugal force yields

(2) $$u_{P}=\frac{x_{P}^{2}\cdot \left(\rho_{P}-\rho_{f}\right)\cdot C\cdot g}{18\cdot \eta_{f}},$$

an expression for the sedimentation velocity along the radial axis valid at sufficiently low Reynolds numbers ($Re_{P}\ll 1$). Particles with small diameters $x_{P}$ and a low solid density $\rho_{P}$ traverse a fluid with viscosity $\eta_{f}$ slower, whereas larger or heavier particles can reach the rotor wall faster. Integrating the Stokes settling velocity (Equation (2)) over the liquid pond depth results in an expression for the settling time $t_{\mathrm{sed}}$ of a particle. Substituting $t_{\mathrm{res}}$ with $t_{\mathrm{sed}}$ in Equation (1) leads to an approximated settling distance

(3) $$l=\frac{r_{b}\cdot \ln\left(\frac{r_{b}}{r_{w}}\right)\cdot 18\eta_{f}\cdot {\dot{V}}_{f}}{\pi \left(r_{b}^{2}-r_{w}^{2}\right)\cdot x_{P}^{2}\cdot \left(\rho_{P}-\rho_{f}\right)\cdot C\cdot g}$$

for each particle in a collective where density and size might be distributed over a certain range. Therefore, fractions with sufficiently low settling rates are transported beyond the rotor weir since $l=f\left(x_{P},\rho_{P},\ldots \right)\geq L$. Note that Equation (3) depicts the process in a streamlined manner because it is, in addition to the previous mentioned assumptions, based on the simplification that every particle is introduced at the inlets liquid surface. Moreover, the fluid is considered pre-accelerated in the inlet zone and radial turbulent back mixing is also neglected. Nonetheless, Equation (3) clarifies important influencing parameters of nanoparticle fractionation on the basis of which the results of this paper are discussed.

### 2.2. Spectroscopic Multi-Component Analysis

Originated in the field of analytical chemistry, spectroscopic model approaches are used to correlate an optical signal with either chemical or physical properties of a sample. In UV/vis spectroscopy, the true numerical output of a hardware sensor, analyzing a suspension where *n* dispersed components are embedded in a nonabsorbing medium, is the extinction

(4) $$E_{\lambda }=\log{\left(\frac{I_{0}}{I}\right)}_{\lambda }=\frac{d}{\ln\left(10\right)}\cdot \sum_{n}^{}\alpha_{n,\lambda }.$$

It is defined by the measurable ratio of light intensity before $\left(I_{0}\right)$ and after the sample $\left(I\right)$ considering that monochromatic light in the wavelength range of 200nm≤λ≤800nm passes through a layer with thickness *d* containing homogeneously distributed particles [53]. Each suspended material with its respective volume fraction $\phi_{n}$ and PSD contributes to the attenuation by absorption and scattering phenomena. In a generalized approximation, an effective extinction cross section per unit volume $C_{V,n,\lambda }$ captures the physical and chemical properties of the bulk resulting in a unique attenuation coefficient

(5) $$\alpha_{n,\lambda }=\phi_{n}\cdot C_{V,n,\lambda }$$

for every nth component [54]. Substituting $\alpha_{n,\lambda }$ in Equation (4) expresses the linear change in extinction

(6) $$E_{\lambda }=\sum_{n}^{}\left(\phi_{n}\frac{d\cdot C_{V,n,\lambda }}{\ln\left(10\right)}\right)=\sum_{n}^{}\left(\phi_{n}\;k_{n}\right)$$

with increasing or decreasing $\phi_{n}$, assuming otherwise constant optical properties of the bulk suspension. Expanding Equation (6) to *p* analytic wavelengths enables the determination of *n* concentrations in multi-component systems with one spectrum. However, this requires knowledge of a corresponding set of extinction coefficients $k_{\lambda ,n}$, which are not directly specifiable for arbitrary suspensions. Hence, multivariate regression models are used in a practical environment to estimate these proportionality factors by conducting a calibration procedure. The theoretical background of practical spectroscopic multi-component analysis summarized below is covered in detail in the literature [55,56]. Here, an inverse calibration is proposed in which the concentrations are calibrated to the extinction at several wavelengths. In this approach, the spectroscopic information $E_{\lambda }$ in the evaluated λ-range lose their theoretical background, but the statistics remain present. This enables the practical construction of regression models, which describe the relationship between the targeted concentration of a sample and its unique UV/vis spectrum.

The general concept is as follows: spectroscopic extinction data of *m* calibration standards at *p* wavelengths containing *n* different solids are structured in a $\left(m\times p+1\right)$-dimensional matrix X. To complete the system of linear equations, the $\left(p+1\times m\right)$-dimensional coefficient matrix β is multiplied with X to receive an expression for the $\left(m\times n\right)$-dimensional target matrix

(7) Y=Xβ

here shown in matrix notation. Note that a nonzero intercept fit requires the addition of vector *u* = [1,1,⋯,1] with size $\left(1\times p\right)$ to the extinction matrix and an extra column of regression parameters in β. During calibration, each individual extinction spectrum X = ${\left[E_{1m},\cdots ,E_{pm}\right]}^{T}$ is labeled with the known target concentrations Y = ${\left[\phi_{m1},\cdots ,\phi_{mn}\right]}^{T}$. Using the generalized least squares (OLS) approach, the best possible solution for β in overdetermined systems $\left(p>n\right)$ is given by

(8) $$\begin{array}{c} \beta ={\left(X^{T}X\right)}^{-1}X^{T}Y \end{array}$$

with $X^{T}$ being the pseudo-inverse of extinction matrix X. A quantitative prediction of the concentration vector $\hat{Y}$ = ${\left[{\hat{\phi }}_{1},\cdots ,{\hat{\phi }}_{n}\right]}^{T}$ of a single new sample is performed by the matrix multiplication of the corresponding spectrum $\hat{X}$ = ${\left[{\hat{E}}_{1},\cdots ,{\hat{E}}_{p}\right]}^{T}$ and the converged values in β:

(9) $$\begin{array}{c} \hat{Y}=\hat{X}\beta . \end{array}$$

## 3. Materials and Methods

### 3.1. Experimental Setup

The following section describes the experimental setup. Both single-component and mixed suspensions of a light and heavy particle system were processed in a tubular centrifuge in order to investigate the soft sensors efficiency by determining the particle concentration at the overflow weir. First, we present all substances including their physical properties and the preparation. Then the process layout and experimental procedure will be explained in detail. The last subsection will elaborate on specific quantification methods used to evaluate the separation process.

#### 3.1.1. Particle Systems

Two materials were considered for the continuous evaluation of separation efficiency in multi-component density fractionation. The first experimental product is zinc oxide (ZnO) (Merck KGaA, Darmstadt, Germany), dispersed in demineralized water. The solids’ density is 5610 kg/m³. It crystallizes in the wurtzite structure and small particles appear white due to refraction of the incident light. With its unique optical and electrical properties, the semiconductor ZnO is used in the fabrication process of solar cells, gas sensors and other photo-electronic or acousto-optic devices [57,58,59]. A listing of further known applications, for example, its use as a thermal-conductive filler in the rubber industry or as a UV-radiation absorbent added in pharmaceutical and cosmetic products, can be found in this overview [60]. Prepared in its initial suspension, the primary particles have a diameter of roughly 40 nm, are unstable and tend to form aggregates and agglomerates. Breakage of the agglomerates was achieved with a Sonifier 450D, manufactured by Branson Ultrasonics Corporation. After dilution with demineralized water, a two liter suspension batch was pumped through a cross flow cell. The setup enabled continuous sonification of the ZnO clusters. After two passes, the concentrated suspension was stabilized with 0.1 mM sodium hexametaphosphate (Na6P6O18) (Sigma-Aldrich, St. Louis, MO, USA) to inhibit re-agglomeration. The concentration of stabilizing agent was kept constant during consecutive dilutions in the feed preparation process. The second material is polymethylmethacrylate (PMMA) with a density of 1193 kg/m³. The spherical acrylic glass particles were harvested as a side product in the PLEXIGLAS® production (Evonik Industries AG, Hanau, Germany) and dispersed in demineralized water with a high solids content up to 10 v%. Due to their low density and spherical shape, these nanoparticles are a suitable model system for biological materials such as cells, viruses and small bacteria [33]. As with the ZnO suspensions, PMMA feed and mixtures were prepared with 0.1 mM sodium hexametaphosphate. The morphology of both particle systems is further elucidated by scanning electron microscopy (SEM) analysis given as supplementary information (SI). Moreover, suspension stability is also addressed and listed as SI (Supplementary Materials).

**Figure 2:**
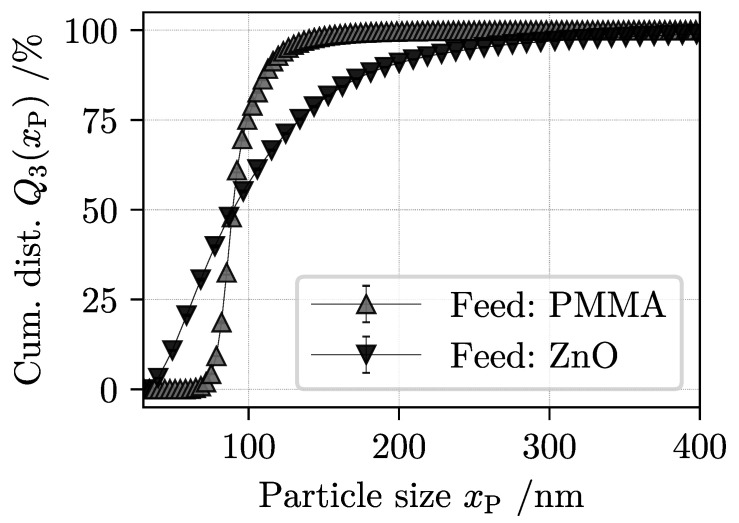
Particle size distribution of single-component suspensions measured with an analytical disk centrifuge.

The volume weighted PSD of both materials shows Figure 2. Particle size was measured by a CPS 24,000 disk centrifuge (CPS Instruments Inc., Prairieville, LA, USA).

After pre-calculation of the equivalent Stokes diameter $x_{P}$, the analytical centrifuge software translates a measured extinction at λ = 470 nm into a relative weight of individual size fractions $\mu \left(x_{P}\right)$ using Mie’s theory [61]. The sample is injected into a density gradient inside a rotating disk. This gradient stabilizes the sedimentation as different particle fractions travel at their respective settling speeds. Near the disks bottom, the laser measures a temporal change in attenuation and subsequently calculates the particle concentration. A resulting distribution of relative weight $\mu (x_{P})$ per particle size class is then used to approximate the sample’s PSD. Key assumptions, however, include that all particles are spheres and have a uniform, constant density and a defined refractive index. Reviewing the distribution of both feed materials shows that the mean particle diameter is nearly identical. In direct comparison, the ZnO particles have a broader distribution with higher proportions of both fine and coarser particles. This can be explained with the above-mentioned, initiatory de-agglomeration where agglomerates are broken down into primary particles and small aggregates of different size. The distribution of PMMA is narrow and particles do not tend to form agglomerates. The preliminary suspension treatment served the purpose of ensuring that both the light and heavy material are in a similar size range.

#### 3.1.2. Process Layout

**Figure 3:**
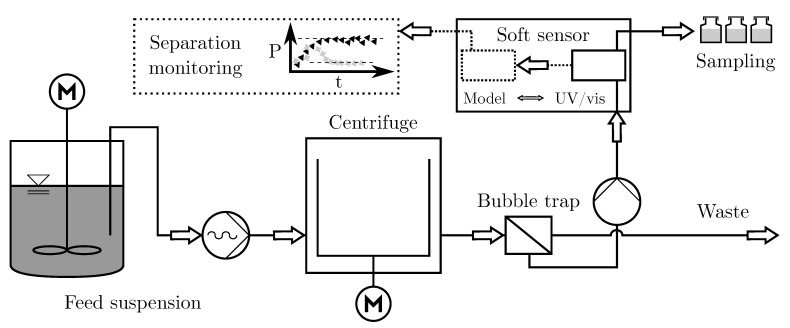
Schematic flow sheet of the monitored separation process.

The experimental setup (Figure 3) includes a Z11-type tubular centrifuge manufactured by Carl Padberg Zentrifugenbau GmbH (CEPA) (Lahr, Germany). The rotor is operable up to a centrifugal number of C = 70,000, which corresponds to a maximum rotor speed of 53,400 min${}^{-1}$. The filling volume of the liquid pond is about 250 mL. Particle separation proceeds according to the principle shown in Figure 1. During operation, an eccentric screw conveyor (NETZSCH Pumpen and Systeme GmbH, Waldkraiburg, Germany) pumps the feed suspension with a constant volumetric flow rate of 100 mL min${}^{-1}$ into the separation zone. A stirred tank contained up to 30 L of pretreated suspension.

The separated fine fraction passes over the overflow weir into a collection tray and is ejected irregularly into the process downstream. A bubble trap separates the suspension–air mixture into a product and waste stream. The fine material, freed from micron sized bubbles, is fed into the UV/vis hardware sensor with a peristaltic pump and a volumetric flow rate of 60 mL min${}^{-1}$. Sampling takes place at the sensor outlet with no dead time in relation to spectral data acquisition. The sensor hardware (Ocean Insight former Ocean Optics, Orlando, FL, USA) used to enable high-speed multi-wavelength extinction measurements consists of a Flame S-XR1 UV/vis spectrometer, a deuterium-halogen DH-2000 light source and a set of optical fibers, which guide the light to both a cross flow cell and back to the detector.

**Figure 4:**
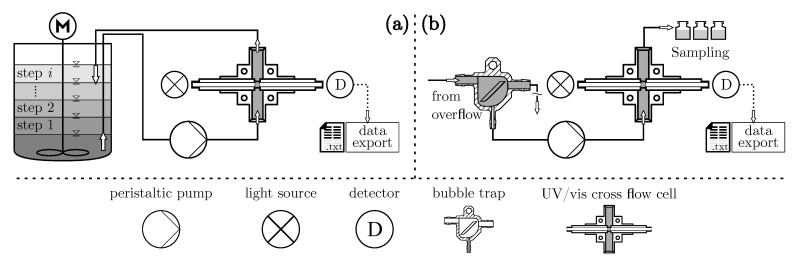
Illustration of the calibration setup (**a**) and the experimental setup (**b**) of the hardware sensor. Components are depicted and named in the bottom legend.

Between two fused silica windows, monochromatic light passes a one millimeter thick suspension layer and becomes attenuated by the excitation of molecules and scattering phenomena induced by dispersed particles. The schematic structure of the sensor is shown in (Figure 4b). Every 400 ms one spectrum is recorded and locally saved as a text file. The extinction $E_{\lambda }$ is measured in a wavelength range of 200 nm ≤λ≤ 800 nm. Each measurement can be assigned to a process time outlining the change in extinction at several wavelengths. Regarding sensor calibration with samples of known concentration, the hardware setup is slightly altered, as outlined in Figure 4a. Here, dispersion is continuously stirred and cycled through the cross flow cell. A pre-calculated and gradual dilution with demineralized water or suspension in multiple steps allows a time efficient acquisition of calibration data sets. This procedure is described more thoroughly in the supplementary material of this paper.

#### 3.1.3. Separation Efficiency Evaluation

When sorting by size and density, two factors are decisive for separation characterization. First, material composition in feed and overflow must be determined efficiently in order to monitor the selectivity in material separation during semi-continuous centrifugation. Given a mixed nanoparticle dispersion containing *n* different solids, the specific product loss

(10) $$P_{n}=\frac{\phi_{n,\mathrm{weir}}}{\phi_{n,\mathrm{feed}}}$$

of the *n*th substance is defined as the portion of their respective content in the weir and feed stream. If no deposition of solids occurs at fixed process conditions $\left(C,{\dot{V}}_{f}\right)$, the specific product loss is unity. Depending on the materials density and size, its value decreases if the considered fractions are separated to a larger extend. In the context of this work, the solids volume fraction of individual components was extracted by either a sampling procedure followed by an invasive offline analysis or with the presented online technique involving a non-invasive UV/vis soft sensor trained with calibration data. In the case of one-component systems $\left(n=1\right)$, a fixed volume of multiple suspension samples are dried and weighted in order to determine the solids volume fraction. This can be done for both PMMA and ZnO after sampling at different process times. Note that the weighted dry matter was corrected for the amount of stabilizer that remains in the dried sample. Regarding mixtures of both solids $\left(n=2\right)$, only the total solid mass – removed – can be evaluated with this technique. In order to infer the proportional mass of both PMMA and ZnO after separation, an inductively coupled plasma optical emission spectrometry (ICP-OES) analysis was carried out. The analytical technique measured the content of pure zinc in each sample, which allowed a stoichiometric approximation of the zinc oxide concentration. Since the total amount of solids (TAS) $m_{\mathrm{TAS}}$ is composed of zinc oxide and PMMA under exclusion of the stabilizing agents mass, the nanoplastic content can be quantified with the closing condition as follows:

(11) $$m_{\mathrm{PMMA}}=m_{\mathrm{TAS}}-m_{\mathrm{ZnO}}.$$

ICP-OES data used for the practical estimation of zinc oxide mass are available as SI (Supplementary Materials).

Besides the holistic contemplation of material separation, the grade efficiency

(12) $$T_{n}\left(x_{P}\right)=1-\frac{\mu_{n,\mathrm{weir}}\left(x_{P}\right)}{\mu_{n,\mathrm{feed}}\left(x_{P}\right)}$$

of component *n* is the second quantity to help evaluate the density fractionation experiments. The separation probability is calculated by the ratio of material specific relative mass $\mu_{n}$ per particle size $x_{P}$ in weir and feed samples. In regard to the described measurement principles of the CPS disk centrifuge it was not possible to measure the relative mass of both dispersed materials at the same time. In a preliminary experiment it was observed that ZnO can be successfully stabilized by sodium hexametaphosphate. Moreover, the preliminary examination revealed that a further increase in the stabilizer concentration to 4.5 mM resulted in the complete dissolution of the ZnO particles. Consequently, the samples turbidity vanished and the corresponding extinction spectrum was congruent with the measured background of demineralized water. Crucially, the PMMA NPs are not affected by the increased concentration of the stabilizing agent. Therefore, sodium hexametaphosphate was added to a mixed suspension before analyzing it in the CPS disk centrifuge. The resulting dissolution of ZnO NPs enables an interference-free measurement of the PSD and, therefore, the grade efficiency of the suspended polymer.

### 3.2. Soft Sensor Setup

Key methodologies and guidelines regarding the construction, diagnostic and maintenance of soft sensors that perform regression tasks are listed in two review articles of Kadlec et al. [41] and Souza et al. [42]. Drawing from their given overview and examples, this section will elaborate on the structure of the developed OLS-based soft sensor written in the Python 3 programming language. The software consists of three main parts: data pre-processing, model diagnostic and application. A detailed sequence of performed operations and their description is explained in the following. For basic data manipulation and mathematical operations on vectors and matrices, the array programming package NumPy [62] was used. Subroutines regarding calibration data analysis and regression tasks are implemented by the application programming interface (API) of the machine learning library scikit-learn [63].

#### 3.2.1. Data Pre-Processing

The first input of the model design pipeline is a calibration data set recorded in the setup shown in Figure 4a. It consists of a so-called design matrix X with the dimensions m×p containing only raw extinction data. As described in Section 2.2, there are *m* calibration samples and *p* wavelength dependent extinction values in one spectrum. Additionally, a corresponding variable matrix Y with size m×n is filled with the concentration information $\phi_{n}$ of *n* samples. The strategy behind this pre-processing step was to reduce the size of data matrix X while decreasing the model’s prediction error on independent data at the same time. In literature, this process is referred to as feature selection and seeks to find the most relevant variables in X with the help of a filter method. For this study, a univariate mutual information (MI) statistic was chosen and implemented via the scikit-learn API. It computes the relatedness between each individual signal $X_{p}={\left[E_{p1},E_{p2},\cdots ,E_{\mathrm{pm}}\right]}^{T}$ and response $Y_{n}={\left[\phi_{n1},\phi_{n2},\cdots ,\phi_{\mathrm{nm}}\right]}^{T}$ column-wise with a nearest neighbor method [64]. The algorithm thus points to those wavelengths that are most likely to increase the correlation between the measured data and the target variable, quantifying it with a dimensionless MI index. In light of this, data reduction is performed manually by discarding wavelengths with low MI values, generating an adjusted matrix X with size m×d. To justify this selection, the model is then evaluated based on an intrinsic cross validation method described in the following section.

#### 3.2.2. Model Diagnostic and Application

**Figure 5:**
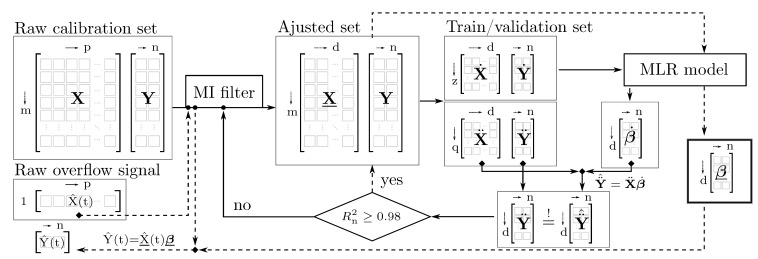
Graphical representation of the sensor software setup described in Section 3.2.1 and Section 3.2.2. Matrices are denoted as brackets with their dimensions drawn on the top and left. The inner loop (solid arrows) highlights the calibration procedure including supervised feature selection with an MI filter, model training and diagnostic measures. The outer loop (dashed arrows) visualizes the translation of raw UV/vis process data $\hat{Y}$(t) into a prediction of the suspension composition $\hat{X}$(t).

The theoretical background given in Section 2.2 established a linear dependence of the measured extinction and the solids volume fraction, which supports the implementation of a straightforward multiple linear regression (MLR) model, solved by the OLS algorithm. Prior to its application in a separation monitoring scenario, an error-estimation technique is needed to determine the intrinsic model quality. In this study, a simple leave-one-out (LOO) cross validation method was pursued. Calibration samples with even index numbers i=0,2,…,z construct the training matrices $\dot{X}$ and $\dot{Y}$ estimating a best fit coefficient matrix $\dot{\beta }$ according to Equation (8). Extinction values of the other odd numbered samples with index j=1,3,…,q fill up validation matrix $\ddot{X}$ used in conjunction with $\dot{\beta }$ to calculate one response vector ${\hat{\ddot{Y}}}_{n}={\left[\phi_{n1},\cdots ,\phi_{\mathrm{nq}}\right]}^{T}$ for each component *n* based on Equation (9). The goodness of fit can be quantified by the coefficient of determination

(13) $$R_{n}^{2}=1-\frac{\sum_{j=1}^{q}{\left({\ddot{\phi }}_{n,j}-{\hat{\ddot{\phi }}}_{n,j}\right)}^{2}}{\sum_{j=1}^{q}{\left({\ddot{\phi }}_{n,j}-{\bar{\ddot{\phi }}}_{n}\right)}^{2}}$$

for each individual component comparing the solids volume fractions ${\ddot{\phi }}_{n,j}$ with the corresponding model prediction ${\hat{\ddot{\phi }}}_{n,j}$. Here, ${\bar{\ddot{\phi }}}_{n}$ denotes the mean of each column in $\ddot{Y}$. Values of $R_{n}^{2}$ close to unity suggest a high prediction strength of the regression model [65]. If the correlation is not satisfactory, X can be adjusted manually in further iterations of the calibration pipeline, as outlined in Figure 5. Lastly, a chosen adjusted calibration set calculates the coefficient matrix β substituted in Equation (9) to quantify the composition $\hat{Y}$(t) of in situ acquired process samples.

## 4. Results

This chapter elaborates on a documented field test of the developed soft sensor used in real-time suspension analysis at the overflow of a tubular centrifuge. The experimental endeavor includes the processing of PMMA in single-component suspension (*expC-1*) and the fractionation of both PMMA and ZnO in a mixture (*expF*). A short-time experiment (*expC-2*), in which a ZnO suspension is processed at C = 10,000, is used for comparative purposes only to analyze the grade efficiency in the CPS disc centrifuge.

An overview of the initial solids volume fractions in the product feeds as well as the corresponding operating parameters is shown in Table 1. Low initial particle concentrations ensure an inferior influence of the sediment build-up on the overflow monitoring. During classification (*expC-1*) at a constant centrifugation number, signal stability and separation efficiency is observed. In fractionation (*expF*) on the other hand, a ramp up in rotor speed is set to monitor changes in multivariate suspension composition under varying process conditions. The soft sensor is set to predict the solids volume fraction ${\hat{\phi }}_{n}$ in an effort to quantify the specific product loss (Equation (10)) in real-time. Unless otherwise stated, separation proceedings were carried out two-fold. Consequently, the predictive sensor output is based on two unique extinction signals per unit of time in the centrifuge overflow. Similarly, lab scale reference measurements on suspension composition are repeated at least three times to obtain a mean and standard deviation. The first part highlights the sensor calibration procedure and model framework used to monitor the separation process, whereas the second part focuses on the soft sensor application and separation outcome evaluation.

### 4.1. Sensor Calibration

**Figure 6:**
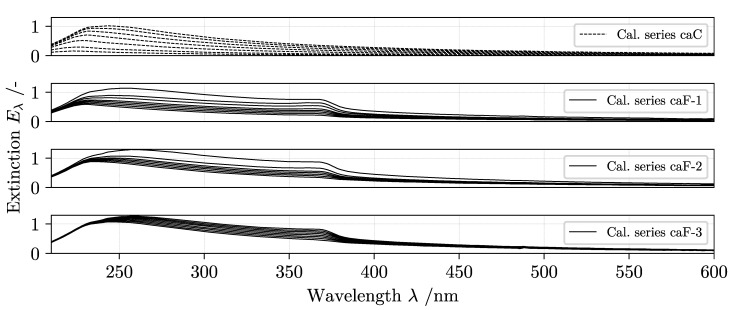
Overview of collected calibration spectra for classification (*caC*) and fractionation (*caF*).

All feed suspensions and calibration mixtures are prepared using the same initial configuration of the particle system PMMA and ZnO. Figure 6 is a graphic summary of the two raw extinction data sets ${\left\{X,Y\right\}}_{\mathrm{caC}}$ and ${\left\{X,Y\right\}}_{\mathrm{caF}}$ acquired in the hardware sensors calibration setup (Figure 4a). A detailed description of the dilution procedure and tabulated concentration data for each spectrum is presented as SI (Supplementary Materials). Note that all spectra of the three lower plots in Figure 6 are combined to form the data set named *caF* used in continuous fractionation monitoring.

The general picture emerging from the spectral analysis is that samples containing dispersed ZnO colloids can be identified based on their unique interactions with the incident light. Studies on the optical properties of ZnO support the hypothesis that an observable extinction peak at λ≈370 nm corresponds to the optical absorption edge of the material [66,67]. Suspensions containing solely PMMA nanoparticles show comparable attenuation in the UV range but interact less in the visible light range without any additional characteristic peak. In an ideal case, the information of an analyte is isolated at specific wavelengths with no interference. In applied UV/vis monitoring, however, both scattering phenomena and absorbance spectra of other suspended components limit the selectivity of a given analytical model due to cross-sensitivities and spectral overlap [55]. In an effort to reduce these interferences computationally, the raw calibration spectra are processed based on the principle shown in Figure 5.

**Figure 7:**
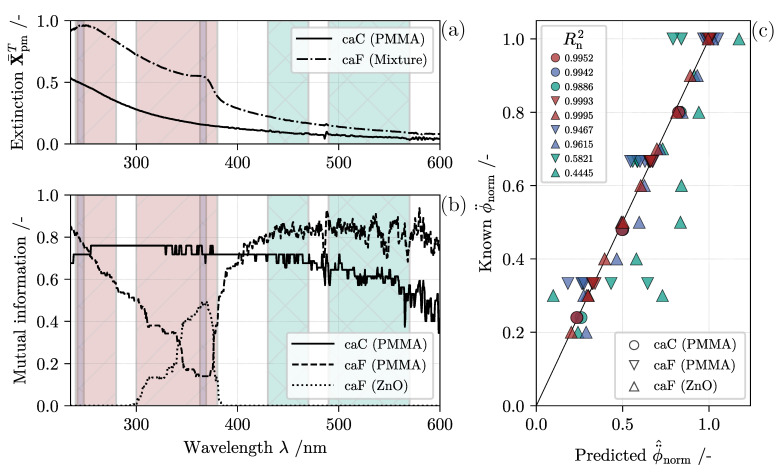
Outcome of applied data pre-processing and model diagnostic. On the left in the background, three selected data segmentation zones are displayed: feature range FR1 (blue, hatched sideways from top to bottom), FR2 (red, hatched sideways from bottom to top) and FR3 (green, cross hatched). (**a**) Mean extinction of calibration data set *caC* and *caF* drawn over the wavelength range of interest. (**b**) Mutual information for components PMMA and ZnO at every wavelength. (**c**) Visual inspection of model suitability plotting predicted ${\hat{\ddot{\phi }}}_{n,m}$ against known ${\ddot{\phi }}_{n,m}$ concentrations. Data points are normalized to the highest volume fraction in each column of matrix $\ddot{Y}$. For every component, the coefficient of determination $R_{n}^{2}$ is displayed.

Preliminary results on calibration data pre-processing are documented in Figure 7, outlining the applied univariate MI computation at every wavelength in the bottom left diagram. The solid line indicates the MI score analyzing data set *caC*, whereas the two dashed and dotted lines correspond to the information gain at λ = *p* in data set *caF* with regard to both dispersed material concentrations separately. For both sets, significant fluctuations in the MI index at higher wavelengths are observable. As illustrated in Figure 7a by the averaged spectra of calibration set *caC* and *caF*, this can be explained by a decreasing signal-to-noise ratio in the high λ-range. For *n* = 1 the MI statistic shows a good relatedness between the solids volume fraction $\phi_{\mathrm{PMMA}}$ and the measured extinction in the range 225 nm ≤λ≤ 600 nm. This observation is plausible for single-component suspension since only one material contributes to the measured extinction spectra. The mixed suspensions on the other hand show a different MI pattern, hinting at possible cross-sensitivities in bands where single-component extinction spectra overlap and add up to the recorded data. An intriguing fact is that a peak in the MI between $\phi_{\mathrm{ZnO}}$ and the extinction is present around λ≈ 370 nm, which underlines the characteristic ZnO absorption edge once again. Based on this, it is advisable to include these wavelengths to the multivariate regression model in order to predict $\phi_{\mathrm{ZnO}}$ with the best possible error reduction. In the case of data set *caC*, however, the MI statistic implies that all wavelengths share a similar amount of valuable information.

To validate this statement, three different evaluation ranges are applied, reducing the available size of the data matrices X. The resulting ranges are illustrated in Figure 7a,b. The first feature range (FR1) is the smallest but centers on two areas with a high MI index. Likewise, the second range (FR2) targets the same extinction bands but extends the included wavelengths, constructing a larger design matrix. Lastly, the third feature range (FR3) has the exact same size as FR2 but the area is shifted towards lower energy frequencies where the information gain in regard to ZnO equals zero and the signal to noise ratio is reduced. Hence, for this λ-range, the target $\phi_{\mathrm{ZnO}}$ is independent from the estimator variable.

Nevertheless, all ranges were used separately for both calibration data sets in the inner validation loop outlined in Figure 5 to diagnose the model’s suitability. Error estimation is visualized by plotting the predicted versus known concentrations in Figure 7c and displaying the coefficient of determination calculated with Equation (13) for both databases and components. For improved readability of the diagnostic, volume fractions are normalized to the highest $\phi_{n}$ of the respective column in $\ddot{Y}$. Univariate MLR results (circles) validate the expected high prediction strength. However, as anticipated, the reduced signal quality of FR3 causes a slight deterioration in model accuracy. The error for multivariate composition estimation (${\ddot{\phi }}_{\mathrm{PMMA}}$, ${\ddot{\phi }}_{\mathrm{ZnO}}$) is visualized by squares and triangles, respectively. After revision of the corresponding datapoints in Figure 7c, a prominent distinction in model suitability can be made. Model construction at higher wavelengths (FR3) as well as a reduced size of the wavelength range (FR1) with $R^{2}\leq$ 0.96 seem to have a negative effect on intrinsic model quality in comparison to evaluation range FR2 ($R^{2}\geq$ 0.99). In light of this, continuous overflow monitoring is based on MLR models that used a data pretreatment according to FR2. For comparison purposes, the two additional domains are fully analyzed as well, but the respective evaluation plots are removed from the visual representation in the following sections and given as SI (Supplementary Materials).

### 4.2. Separation Outcome

**Figure 8:**
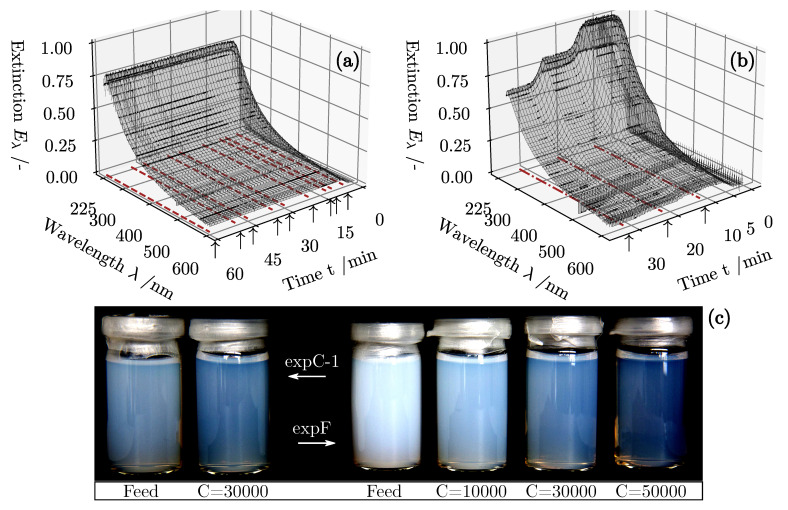
Raw extinction data of experimental series *expC-1* (**a**) and *expF*(**b**) measured with the experimental setup (Figure 4b) of the UV/vis hardware sensor. Arrows and dash dotted lines mark sampling times. (**c**) Collected overflow samples at constant *C*-values and plateaus of extinction. Feed samples visualized for comparison between transmittance before and after centrifugation.

**Figure 9:**
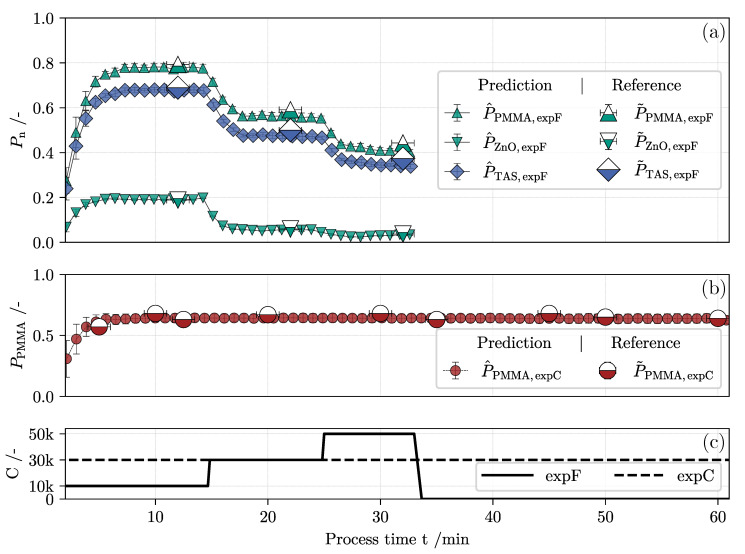
Comparison of sensor output (solid symbols) and laboratory analysis (half-filled symbols) of the product loss in fractionation (**a**) and classification (**b**) monitoring over the elapsed process time. The set centrifugation number is drawn on shared abscissa (**c**). The soft sensor output is based on the corresponding calibration data set of feature range FR2.

Continuing with the second part of the experimental evaluation, Figure 8 highlights the measured raw extinction data during classification (*expC-1*) and fractionation (*expF*). Signal data are laid out in a three-dimensional space, plotting raw extinction against the elapsed process time *t*. Signal stability in the desired evaluation range 225 nm ≤λ≤ 600 nm is high, as bubbles are effectively removed from the sensor inlet by the bubble trap. Initially, demineralized water is displaced by the suspension lowering the transmittance until a steady state is reached. Furthermore, the listed operating parameters in Table 1 are correctly mapped by the recorded course of extinction. Regarding classification of PMMA, the acquired spectra in Figure 8a indicate constant optical properties in the overflow. In comparison, Figure 8b highlights the rotor speed ramp up with three distinct plateaus of constant light attenuation during fractionation. A detailed view of speed level settings against the process time is shown in Figure 9c.

Sampling of roughly 200 mL of fines takes place in a period of two minutes at the sensor outlet. Each individual time of extraction is marked with small arrows and red dash dotted lines. A reference is only taken when the system is in a steady state indicated by a constant UV/vis signal. Four of these samples and their corresponding feed dispersions are shown in the lower half of Figure 8. Qualitative inspection with the human eye shows a stronger turbidity in mixed suspension, caused by the dispersed ZnO particles and their high refractive index. Overall, the transmittance increases with rotor speed due to more solids being deposited at the rotor wall.

#### 4.2.1. Evaluation of Size Fractionation

**Figure 10:**
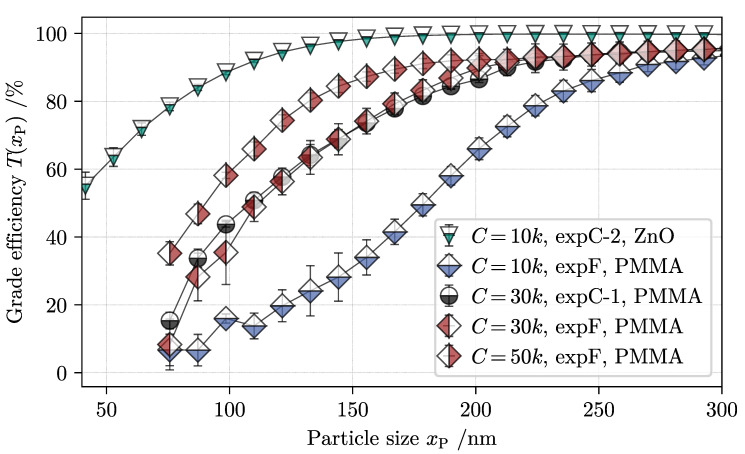
Summary of grade efficiency plots for PMMA after fractionation at three C-values (diamonds) and classification at a constant rotor speed (circles). An additional classification run of a pure ZnO suspension at C = 10,000 yields the leftmost partition curve (triangles).

After analyzing the weir and feed samples in the CPS disc centrifuge, Equation (12) provides the necessary data to reveal the separation efficiency of the apparatus in Figure 10. Diamonds indicate the partition curves of PMMA in fractionation for the set C-value ramp whereas circles mark the grade efficiency of PMMA after classification (*expC*). At this point the reader is reminded that the described procedure of analytical centrifugation is only applicable in single-component systems, as a material specific adjustment to the device is needed beforehand. In light of this, samples taken from *expF* are pretreated with additional stabilizing agent until ZnO nanoparticles are completely dissolved. Accordingly, only the separation curves of the polymer particles are shown for fractionation experiment analysis. The evaluated partition curves of *expC-1* and *expF* are congruent, which confirms two things. First, by dissolving ZnO before the particle size analysis, a valid and reproducible approximation for the grade efficiency of PMMA NPs is feasible. Second, ZnO NPs at such low concentrations do not affect the sedimentation of lighter polymer particles during centrifugation in a tubular centrifuge.

As expected according to fundamentals, an increase in rotor speed leads to a noticeable shift of the partition curves to smaller particle sizes. At C = 10,000, the median cut size $x_{T,50\%}$ is 180 nm, whereas at C = 50,000, the same cut size equals 90 nm for PMMA NPs. Furthermore, the comparative result of *expC-2* shows that higher particle density leads to smaller cut sizes at constant operating conditions. More specifically, ZnO nanoparticles with a diameter larger than 150 nm are completely retained in the centrifuge rotor, whereas the separation efficiency for same size polymer particles at C = 10,000 is only around *T*(150 nm) = 30%.

In summary, analytical centrifugation supports the hypothesis that tubular centrifuges can simultaneously sort particles according to their size and material density. Up to this point, however, the separation degree of the metal oxide is only validated by the additional classification *expC-2*. Unfortunately, the same analytic technique cannot be applied to mixtures of ZnO and PMMA in the CPS disc centrifuge. Hence, a supportive methodology is needed to quantify the separation efficiency of heavier particles in multi-component suspension processing. For this purpose and for continuous overflow monitoring, suspension composition is predicted by the constructed soft sensor.

#### 4.2.2. Continuous Monitoring of Fine Fraction Composition

Referring to the outlined principle of sensor training and overflow monitoring in Figure 5, every 400 ms, a raw extinction spectrum $\hat{X}$(t) is reduced to an adjusted vector $\hat{X}$(t) and multiplied by the corresponding predictor β. Using Equation (9) the overflow composition

Y^(t)=ϕ^PMMA(t),ϕ^ZnO(t)T=X−^(t)β−

is evaluated in real-time with low computational effort. Note that each calibration data set provides a unique coefficient matrix β tailored to the online monitoring of either *expC-1* or *expF*. Knowing the feeds’ relative concentration, a specific product loss ${\hat{P}}_{n}$ is then calculated with Equation (10) for every component. In Figure 9, an overview of the predicted product loss (small symbols) is given for both classification and fractionation experiments on the y-axis. Here, raw extinction data were reduced according to feature range FR2. Prediction results of domains FR1 and FR3 are visualized in two similar plots in the SI (Supplementary Materials). The abscissa is shared between the subfigures and shows the elapsed process time. For de-cluttering purposes, every 15th datapoint is shown. Furthermore, each individual marker is displaced 1.42 minutes to the left due to the observed dead time between the centrifuge outlet and sensor inlet. Superposed large symbols represent the offline reference ${\tilde{P}}_{n}$. Further details on the laboratory analysis performed to determine the relative solids volume fraction in each sample, and thus the corresponding true product loss, can be found in Section 3.1.3. Horizontal error bars symbolize the time it took to gather the samples.

In the following, ${\hat{P}}_{n}$ and ${\tilde{P}}_{n}$ of each component are discussed individually based on the introduced theory of nanoparticle separation written in Section 2.1. Contemporaneously, the sensor performance is specified by the mean prediction error (MPE):

(14) $${\mathrm{MPE}}_{n}=\frac{\left|{\tilde{\phi }}_{n}-{\hat{\phi }}_{n}\right|}{{\tilde{\phi }}_{n}}\times 100\%$$

and summarized for each individual feature range and material in Table 2.

In Figure 9b, the sensor translates unseen extinction spectra (Figure 8a) into the corresponding product loss of PMMA during classification. Centrifugation at a C-value of 30,000 results in a constant product loss of around 60%. Predictive and reference measures prove that no significant rise of product loss takes place during classification. This can be explained by the consciously chosen, low feed concentrations. The sediment formed in the rotor does not affect the deposition of the polymer since its volume does not shorten the particles residence time, resulting in a constant separation efficiency.

The determined product loss of all samples taken during this time period coincides with the values generated by the MLR predictor with an MPE of just 5.913%. The other feature ranges perform slightly worse, although the inclusion of wavelengths where extinction shows a lower signal-to-noise ratio seems counter-intuitive. Rather, it is more reasonable to evaluate spectral data at wavelengths where most of the chemical information is located in interference-free signals. This is the case for FR2 incorporating multiple wavelengths with high MI indices.

Assuming that random errors in the recording of the spectra during processing or analysis errors of the reference measurements are negligible, a systematic error can be observed. With few exceptions, the soft sensor output tends to underestimate the true product loss in the centrifuge overflow. These deviations can be explained by the attenuation coefficient expressed in Equation (5). Therein, both absorption and scattering cross sections define $C_{V,n,\lambda }$ as an optical constant of the analyzed suspension. As highlighted in Figure 10, PSDs of both materials are adjusted during separation, emphasizing a possible change in the effective extinction cross section of the bulk suspension. Strictly speaking, the linear relationship defined in Equation (6), therefore, no longer applies to the in situ extinction analysis of the fine fraction samples. According to fundamentals, the wavelength dependent scattering intensity is altered by the materials refractive index and scales with particle size to the power of six [68]. Hence, the induced separation of the coarser fraction in classification and fractionation affects the shape of a measured extinction spectrum. Yet, the MPE regarding the online monitoring of PMMA classification is low although the systems PSD is altered. One explanation is that differences in the mean volume weighted diameter of the feed (103 nm ± 1 nm) and the overflow samples (89 nm ± 0.5 nm) are very small. This leads towards the assumption that the scattered light intensity does not change significantly enough to hurt the suitability of the established linear soft sensor model. Similar observations were made during studies establishing a continuous overflow monitoring based on measurements of scattered light [39]. Here, calibration data sets were also recorded from dilutions of the product feed, which failed to sensitize the underlying model with respect to the changes in the materials PSD.

The second experiment to assess is the density fractionation of both PMMA and ZnO in mixture highlighted in Figure 8a. The overall product loss analyzed by gravimetric offline measurements is depicted by half filled, blue diamonds. Note that the TAS here refers to the corrected mass of both PMMA and ZnO in the dried sample excluding the weight of Na6P6O18 crystals. It is clearly shown that the ramp up in rotor speed leads to a better separation of the suspended solids. The sensor can therefore identify changes in the operating parameters with little delay. With Equation (11), this information was used in conjunction with the ICP-OES analysis to compute the true relative solids volume fraction of both materials. In direct comparison, ZnO is separated more effectively at each of the three C-values due to its higher density and faster radial movement in the centrifuge rotor according to Equation (2). Consequently at C= 30,000, 6.0% of the heavy material remains in the product stream. At C= 50,000, mere traces of ZnO are measurable exclusively by the conducted ICP-OES analysis, verifying a product loss of 3.4%. Online monitoring as well as lab scale reference measurements outline reproducible magnitudes of product loss at the three distinct plateaus with constant rotor speed. When reviewing the MSE, a moderate prediction error of 4.698% in the case of the light polymer can be observed, which is comparable to the prediction quality in classification. Regarding the heavy metal oxide ZnO, however, predictions for ${\hat{P}}_{\mathrm{ZnO}}$ underestimate the true solids volume fraction in the overflow by 8.5%. Error discussion jet involves basic theory of light scattering by an ensemble of particles. In the case of ZnO, the feed distribution is broader and therefore the particles effective extinction cross section is accompanied by a more pronounced scattering part. It is likely that the removal of coarse particle fractions during centrifugation results in a considerable alteration of the bulks’ optical properties. Because the sensor software is only capable of modeling linear dependence of extinction and concentration at constant PSD, this effect could explain the more inaccurate predictions of the sensor in regard to the product loss of ZnO. Several studies [69,70] list the impact of changing PSDs as a confounding factor in quantitative spectroscopic analysis of particle-loaded fluids. Possible adjustments to the model may include an empirically determined correction factor that adjusts the sensor output in accordance to the change in scattering properties of the sample. It is also conceivable that a more diverse calibration data set could be recorded based on collected overflow samples at variable operating parameters. The results of a preliminary study highlight the potential benefit in prediction accuracy when incorporating classified samples in the calibration procedure regarding spectroscopic multi-component analysis [43]. The disadvantage here, however, is the increased effort required to calibrate the UV/vis sensor. In light of this, multiple regression at several carefully chosen wavelengths marks a supportive approach presented in this paper. In the future, it is imaginable to evaluate the extinction spectra of samples with known concentrations and PSD to be able to perform more precise estimations of the fine fractions composition.

Taken altogether, the data presented here provide strong evidence that the developed soft sensor is able to monitor the solids volume fraction in the overflow of a tubular centrifuge. Despite error prone predictions regarding the material ZnO, the product loss is monitored qualitatively and plausibly according to induced changes in operating parameters. In the case of PMMA NPs separation, accurate predictions are achievable when following the suggested method (Figure 5) of sensor calibration.

## 5. Conclusions

The demand for polydisperse particle systems in the nanometer range, which have defined properties in regard to their PSD and material composition, is steadily increasing. Tubular centrifuges offer an approach for bench scale classification and fractionation of these particles. Due to their semi-continuous operation mode, however, process monitoring is needed to assist further optimization endeavors. For this reason, the presented study involves a UV/vis soft-sensor, which was installed in the tubular centrifuges overflow. Its hardware part continuously acquired an extinction spectrum of processed fines containing either polymer NPs (PMMA), suspended metal oxide particles (ZnO) or a mixture of both. Furthermore, a multivariate regression model scheme was developed and connected to the raw extinction data processing. For this purpose, a sensor calibration procedure with feed samples of known concentration and material composition was mandatory. Extinction values measured at wavelengths that contributed to the regression model quality were identified and manually separated from statistically insignificant inputs. This enabled parallel, real-time model-based predictions of the solids volume fraction of both processed materials in the centrifuge overflow. When compared to offline sampling and costly laboratory analysis, the model has a low prediction error with respect to the approximation of the solids volume fraction of PMMA. In the case of ZnO, a significantly greater degree of material separation at increasing rotor speeds is observed. Accompanied by a more drastic shift in the PSD, the precise computation of the solids volume fraction of metal oxide NPs was complicated. This was explained by fundamental theory of light scattering by small particles and the influence of the changing extinction cross section due to induced separation of coarser fractions. Continuing studies may include the same soft-sensor setup but follow a different routine in calibration data acquisition. This could successfully improve the model based on a correction factor if inconsistent scattering properties of the analyzed fines are expected. Nonetheless, the presented setup enables the efficient online monitoring of both classification and material sorting in tubular centrifuges at low particle concentrations. Because a fast response to changes in operating parameters was achieved, the soft sensor setup leads to an improved understanding of the separation process and its underlying mechanics. Finally, its integration opens up new perspectives regarding real-time process control for product quality maintenance.

## Figures and Tables

**Table 1:** Listed information on feed suspensions and operating conditions.

			Operating Parameters				
Experiment	Feed Concentration		Volumetric Flow Rate	Centrifugal Number			Process Time
	$\phi_{\mathrm{PMMA}}$/-	$\phi_{\mathrm{ZnO}}$/-	${\dot{V}}_{f}$/$\mathrm{mL}\cdot \min^{-1}$	$C_{1}$/-	$C_{2}$/-	$C_{3}$/-	*t*/min
*expC-1*	$1.359\times 10^{-3}$	0	100	30,000	-	-	60
*expC-2*	0	$9.264\times 10^{-5}$	100	10,000	-	-	15
*expF*	$1.631\times 10^{-3}$	$7.181\times 10^{-5}$	100	10,000	30,000	50,000	35

**Table 2:** Prediction error for the solids volume fraction ϕ specified for each material (PMMA, ZnO) as well as the combined amount of suspended solids (TAS) excluding the mass of stabilizing agent Na6P6O18. The row related to the chosen feature range FR2 has the lowest MPE.

	Mean Prediction Error ${\mathrm{MPE}}_{n}$ /%			
	***expC-1***	***expF***		
**Feature Range**	**PMMA**	**PMMA**	**ZnO**	**TAS**
FR1	10.86	12.42	26.76	11.98
FR2	5.913	4.698	8.499	4.740
FR3	9.06	33.23	800.9	9.059

## Data Availability

The data presented in this study are available on request from the corresponding author.

## Supplementary Materials

The following are available online at https://www.mdpi.com/article/10.3390/nano11051114/s1, Figure S1: Schematic view of the calibration setup, Figure S2: Spectra acquired from four continuous dilution series, Table S1: Solids volume fractions of each sample extracted from the continuous dilution series, Figure S3: Qualitative SEM image analysis, Figure S4: Suspension stability analysis, Table S2: Reference concentration data (Zn, ZnO), Table S3: Reference concentration data (TAS, PMMA), Figure S5: Sensor output evaluation (FR1), Figure S6: Sensor output evaluation (FR3).

## Abbreviations

The following abbreviations are used in this manuscript:

API	application programming interface
ICP-OES	inductively coupled plasma optical emission spectrometry
MI	mutual information
MLR	multiple linear regression
Na6P6O18	sodium hexametaphosphate
NPs	nanoparticles
OLS	ordinary or generalized least squares
PSD	particle size distribution
PMMA	polymethylmethacrylate
SEM	scanning electron microscope
SI	supplementary information
TAS	total amount of solids
UV/vis	ultraviolet-visible
ZnO	zinc oxide

## References

[1] Wong A., Liu Q., Griffin S., Nicholls A., Regalbuto J.R. (2017). Synthesis of ultrasmall, homogeneously alloyed, bimetallic nanoparticles on silica supports. Science.

[2] Alegret N., Criado A., Prato M. (2017). Recent Advances of Graphene-based Hybrids with Magnetic Nanoparticles for Biomedical Applications. Curr. Med. Chem..

[3] Adair J.H., Suvaci E. (2000). Morphological control of particles. Curr. Opin. Colloid Interface Sci..

[4] Zhang J., Yang S., Chen Z., Yan Y., Zhao J., Li J., Jiang Z. (2018). In Situ synthesis of SiC-graphene core-shell nanoparticles using wet ball milling. Ceram. Int..

[5] Malamatari M., Taylor K.M., Malamataris S., Douroumis D., Kachrimanis K. (2018). Pharmaceutical nanocrystals: Production by wet milling and applications. Drug Discov. Today.

[6] Ramos A.P., Cruz M.A.E., Tovani C.B., Ciancaglini P. (2017). Biomedical applications of nanotechnology. Biophys. Rev..

[7] Liu W.T. (2006). Nanoparticles and their biological and environmental applications. J. Biosci. Bioeng..

[8] Zhang X. (2015). Gold Nanoparticles: Recent Advances in the Biomedical Applications. Cell Biochem. Biophys..

[9] Geszke-Moritz M., Moritz M. (2016). Solid lipid nanoparticles as attractive drug vehicles: Composition, properties and therapeutic strategies. Mater. Sci. Eng. C.

[10] Panigrahi S., Basu S., Praharaj S., Pande S., Jana S., Pal A., Ghosh S.K., Pal T. (2007). Synthesis and Size-Selective Catalysis by Supported Gold Nanoparticles: Study on Heterogeneous and Homogeneous Catalytic Process. J. Phys. Chem. C.

[11] Narayanan R., El-Sayed M.A. (2004). Shape-Dependent Catalytic Activity of Platinum Nanoparticles in Colloidal Solution. Nano Lett..

[12] He Z., Zhang Z., Bi S. (2020). Nanoparticles for organic electronics applications. Mater. Res. Express.

[13] Shen W., Zhang X., Huang Q., Xu Q., Song W. (2014). Preparation of solid silver nanoparticles for inkjet printed flexible electronics with high conductivity. Nanoscale.

[14] Bliznyuk V., Ruhstaller B., Brock P.J., Scherf U., Carter S.A. (1999). Self-Assembled Nanocomposite Polymer Light-Emitting Diodes with Improved Efficiency and Luminance. Adv. Mater..

[15] Plüisch C.S., Wittemann A. (2013). Shape-Tailored Polymer Colloids on the Road to Become Structural Motifs for Hierarchically Organized Materials. Macromol. Rapid Commun..

[16] Maneeprakorn W., Malik M.A., O’Brien P. (2010). Developing Chemical Strategies for the Assembly of Nanoparticles into Mesoscopic Objects. J. Am. Chem. Soc..

[17] Wang H., Brandl D.W., Nordlander P., Halas N.J. (2007). Plasmonic Nanostructures: Artificial Molecules. Accounts Chem. Res..

[18] Cheon J.Y., Kim S.J., Rhee Y.H., Kwon O.H., Park W.H. (2019). Shape-dependent antimicrobial activities of silver nanoparticles. Int. J. Nanomed..

[19] Suchomel P., Kvitek L., Prucek R., Panacek A., Halder A., Vajda S., Zboril R. (2018). Simple size-controlled synthesis of Au nanoparticles and their size-dependent catalytic activity. Sci. Rep..

[20] Tong S., Quinto C.A., Zhang L., Mohindra P., Bao G. (2017). Size-Dependent Heating of Magnetic Iron Oxide Nanoparticles. ACS Nano.

[21] Woźniak A., Malankowska A., Nowaczyk G., Grześkowiak B.F., Tuśnio K., Słomski R., Zaleska-Medynska A., Jurga S. (2017). Size and shape-dependent cytotoxicity profile of gold nanoparticles for biomedical applications. J. Mater. Sci. Mater. Med..

[22] Cao S., Tao F.F., Tang Y., Li Y., Yu J. (2016). Size- and shape-dependent catalytic performances of oxidation and reduction reactions on nanocatalysts. Chem. Soc. Rev..

[23] Patsula V., Moskvin M., Dutz S., Horák D. (2016). Size-dependent magnetic properties of iron oxide nanoparticles. J. Phys. Chem. Solids.

[24] Adams C.P., Walker K.A., Obare S.O., Docherty K.M. (2014). Size-Dependent Antimicrobial Effects of Novel Palladium Nanoparticles. PLoS ONE.

[25] Zhang S., Li J., Lykotrafitis G., Bao G., Suresh S. (2009). Size-Dependent Endocytosis of Nanoparticles. Adv. Mater..

[26] Plüisch C.S., Bössenecker B., Dobler L., Wittemann A. (2019). Zonal rotor centrifugation revisited: New horizons in sorting nanoparticles. RSC Adv..

[27] Sun X., Tabakman S., Seo W.S., Zhang L., Zhang G., Sherlock S., Bai L., Dai H. (2009). Separation of Nanoparticles in a Density Gradient: FeCo@C and Gold Nanocrystals. Angew. Chem. Int. Ed..

[28] Fagan J.A., Becker M.L., Chun J., Nie P., Bauer B.J., Simpson J.R., Hight-Walker A., Hobbie E.K. (2008). Centrifugal Length Separation of Carbon Nanotubes. Langmuir.

[29] Novak J.P., Nickerson C., Franzen S., Feldheim D.L. (2001). Purification of Molecularly Bridged Metal Nanoparticle Arrays by Centrifugation and Size Exclusion Chromatography. Anal. Chem..

[30] Spelter L.E., Meyer K., Nirschl H. (2012). Screening of Colloids by Semicontinuous Centrifugation. Chem. Eng. Technol..

[31] Lohse S.E., Eller J.R., Sivapalan S.T., Plews M.R., Murphy C.J. (2013). A Simple Millifluidic Benchtop Reactor System for the High-Throughput Synthesis and Functionalization of Gold Nanoparticles with Different Sizes and Shapes. ACS Nano.

[32] Segets D., Komada S., Butz B., Spiecker E., Mori Y., Peukert W. (2013). Quantitative evaluation of size selective precipitation of Mn-doped ZnS quantum dots by size distributions calculated from UV/Vis absorbance spectra. J. Nanopart. Res..

[33] Spelter L.E., Steiwand A., Nirschl H. (2010). Processing of dispersions containing fine particles or biological products in tubular bowl centrifuges. Chem. Eng. Sci..

[34] Spelter L.E., Nirschl H. (2010). Classification of Fine Particles in High-Speed Centrifuges. Chem. Eng. Technol..

[35] Konrath M., Brenner A.K., Dillner E., Nirschl H. (2015). Centrifugal classification of ultrafine particles: Influence of suspension properties and operating parameters on classification sharpness. Sep. Purif. Technol..

[36] Konrath M., Gorenflo J., Hübner N., Nirschl H. (2016). Application of magnetic bearing technology in high-speed centrifugation. Chem. Eng. Sci..

[37] Kohsakowski S., Seiser F., Wiederrecht J.P., Reichenberger S., Vinnay T., Barcikowski S., Marzun G. (2019). Effective size separation of laser-generated, surfactant-free nanoparticles by continuous centrifugation. Nanotechnology.

[38] Flegler A., Schneider M., Prieschl J., Stevens R., Vinnay T., Mandel K. (2016). Continuous flow synthesis and cleaning of nano layered double hydroxides and the potential of the route to adjust round or platelet nanoparticle morphology. RSC Adv..

[39] Konrath M., Hackbarth M., Nirschl H. (2014). Process monitoring and control for constant separation conditions in centrifugal classification of fine particles. Adv. Powder Technol..

[40] Frank U., Wawra S.E., Pflug L., Peukert W. (2019). Multidimensional Particle Size Distributions and Their Application to Nonspherical Particle Systems in Two Dimensions. Part. Part. Syst. Charact..

[41] Kadlec P., Gabrys B., Strandt S. (2009). Data-driven Soft Sensors in the process industry. Comput. Chem. Eng..

[42] Souza F.A., Araújo R., Mendes J. (2016). Review of soft sensor methods for regression applications. Chemom. Intell. Lab. Syst..

[43] Winkler M., Sonner H., Gleiss M., Nirschl H. (2020). Fractionation of ultrafine particles: Evaluation of separation efficiency by UV–vis spectroscopy. Chem. Eng. Sci..

[44] Rhein F., Scholl F., Nirschl H. (2019). Magnetic seeded filtration for the separation of fine polymer particles from dilute suspensions: Microplastics. Chem. Eng. Sci..

[45] Paramelle D., Sadovoy A., Gorelik S., Free P., Hobley J., Fernig D.G. (2014). A rapid method to estimate the concentration of citrate capped silver nanoparticles from UV-visible light spectra. Analyst.

[46] Liu F.K., Ko F.H., Huang P.W., Wu C.H., Chu T.C. (2005). Studying the size/shape separation and optical properties of silver nanoparticles by capillary electrophoresis. J. Chromatogr. A.

[47] Shah D., Wang J., He Q.P. (2019). A feature-based soft sensor for spectroscopic data analysis. J. Process. Control..

[48] Rüdt M., Vormittag P., Hillebrandt N., Hubbuch J. (2019). Process monitoring of virus-like particle reassembly by diafiltration with UV/Vis spectroscopy and light scattering. Biotechnol. Bioeng..

[49] Bartosiak M., Giersz J., Jankowski K. (2019). Analytical monitoring of selenium nanoparticles green synthesis using photochemical vapor generation coupled with MIP-OES and UV–Vis spectrophotometry. Microchem. J..

[50] Rato T.J., Reis M.S. (2018). Building Optimal Multiresolution Soft Sensors for Continuous Processes. Ind. Eng. Chem. Res..

[51] Hendel T., Wuithschick M., Kettemann F., Birnbaum A., Rademann K., Polte J. (2015). Correction to In Situ Determination of Colloidal Gold Concentrations with UV–Vis Spectroscopy: Limitations and Perspectives. Anal. Chem..

[52] Sinn T., Flegler A., Wolf A., Stübinger T., Witt W., Nirschl H., Gleiß M. (2020). Investigation of Centrifugal Fractionation with Time-Dependent Process Parameters as a New Approach Contributing to the Direct Recycling of Lithium-Ion Battery Components. Metals.

[53] Mäntele W., Deniz E. (2017). UV—VIS absorption spectroscopy: Lambert-Beer reloaded. Spectrochim. Acta Part A.

[54] Bohren C.F., Huffman D.R. (2004). Absorption and Scattering of Light by Small Particles.

[55] Otto M.V. (2017). Chemometrics: Statistics and Computer Application in Analytical Chemistry.

[56] Maris M.A., Brown C.W., Lavery D.S. (1983). Nonlinear multicomponent analysis by infrared spectrophotometry. Anal. Chem..

[57] Senoussaoui N., Krause M., Müller J., Bunte E., Brammer T., Stiebig H. (2004). Thin-film solar cells with periodic grating coupler. Thin Solid Film..

[58] Pizzini S., Buttá N., Narducci D., Palladino M. (1989). Thick Film ZnO Resistive Gas Sensors: Analysis of Their Kinetic Behavior. J. Electrochem. Soc..

[59] Müller J., Weißenrieder S. (1994). ZnO-thin film chemical sensors. Fresenius’ J. Anal. Chem..

[60] Kołodziejczak-Radzimska A., Jesionowski T. (2014). Zinc Oxide—From Synthesis to Application: A Review. Materials.

[61] Mie G. (1908). Beiträge zur Optik trüber Medien, speziell kolloidaler Metallösungen. Ann. Der Phys..

[62] Harris C.R. (2020). Array programming with NumPy. Nature.

[63] Buitinck L., Louppe G., Blondel M., Pedregosa F., Mueller A., Grisel O., Niculae V., Prettenhofer P., Gramfort A., Grobler J. (2013). API design for machine learning software: Experiences from the scikit-learn project. arXiv.

[64] Ross B.C. (2014). Mutual Information between Discrete and Continuous Data Sets. PLoS ONE.

[65] Renaud O., Victoria-Feser M.P. (2010). A robust coefficient of determination for regression. J. Stat. Plan. Inference.

[66] Yoshikawa H., Adachi S. (1997). Optical Constants of ZnO. Jpn. J. Appl. Phys..

[67] Srikant V., Clarke D.R. (1997). Optical absorption edge of ZnO thin films: The effect of substrate. J. Appl. Phys..

[68] Seinfeld J.H.V. (2016). Atmospheric Chemistry and Physics: From Air Pollution to Climate Change.

[69] Gippel C.J. (1995). Potential of turbidity monitoring for measuring the transport of suspended solids in streams. Hydrol. Process..

[70] Eerdenbrugh B.V., Alonzo D.E., Taylor L.S. (2011). Influence of Particle Size on the Ultraviolet Spectrum of Particulate-Containing Solutions: Implications for In-Situ Concentration Monitoring Using UV/Vis Fiber-Optic Probes. Pharm. Res..

