# Supplementary material for: Soft Sensor Development for Real-Time Process Monitoring of Multidimensional Fractionation in Tubular Centrifuges
Source: Nanomaterials (Basel). 2021 Apr 25;11(5):1114. doi: 10.3390/nano11051114 (PMC8145064; doi:10.3390/nano11051114)
Supplement: Supplementary file 1 [file nanomaterials-11-01114-s001.zip › nanomaterials-1164422-supplementary.pdf]

Soft Sensor Development for Real-time Process Monitoring of Multidimensional Fractionation in Tubular Centrifuges: Supplementary Material

Marvin Winkler[1], Marco Gleiss[1], and Hermann Nirschl[1]

[1]**Institute of Mechanical Process Engineering and Mechanics, Karlsruhe Institute of Technology (KIT)**

ABSTRACT

High centrifugal acceleration and throughput rates of tubular centrifuges enable the solid-liquid size separation and fractionation of nanoparticles on a bench scale. Nowadays, advantageous product properties are defined by precise specifications regarding particle size and material composition. Hence, there is a demand for innovative and efficient downstream processing of complex particle suspensions. With this type of centrifuge working in a semi-continuous mode, an online observation of the separation quality is needed for optimization purposes. To analyze the composition of fines in the downstream of the centrifuge, a UV/vis soft sensor is developed to monitor the sorting of polymer and metal oxide nanoparticles by their size and density. By spectroscopic multi-component analysis, a measured UV/vis signal is translated into a model based prediction of the relative solids volume fraction of the fines. High signal stability and an adaptive but mandatory calibration routine enable the presented setup to accurately predict the products composition at variable operating conditions. It is outlined how this software-based UV/vis sensor can be utilized effectively for challenging real-time process analytics in multi-component suspension processing. The setup provides insight into the underlying process dynamics and assists in optimizing the outcome of separation tasks on the nanoscale.

Keywords: solid-liquid separation; multidimensional particle features; tubular centrifuge; process monitoring; soft sensor; UV/vis; chemometrics

1 UV/VIS SENSOR CALIBRATION PROCEDURE

In the following, feed suspension processing in the calibration setup of the soft sensor is described in detail. It served the purpose of efficient and fast acquisition of calibration spectra with known particle concentration and material composition.

1.1 Recording of dilution series caC

The calibration dataset for continuous monitoring of the solids volume fraction of polymethylmethacrylate (PMMA) nanoparticles (NPs) in the centrifuge overflow was recorded in the setup highlighted in S1. The initial suspension was diluted with decriminalized water until a solids volume fraction of $\phi_{\mathrm{PMMA}} = 1.0 \cdot 10^{-3}$ was reached. Afterwards, the dispersion was continuously homogenized inside a beaker on a magnetic stirrer. Additionally, the peristaltic pump moved the suspension in a circular fashion through the UV/vis cross flow cell where one extinction spectrum was recorded every 400 ms.

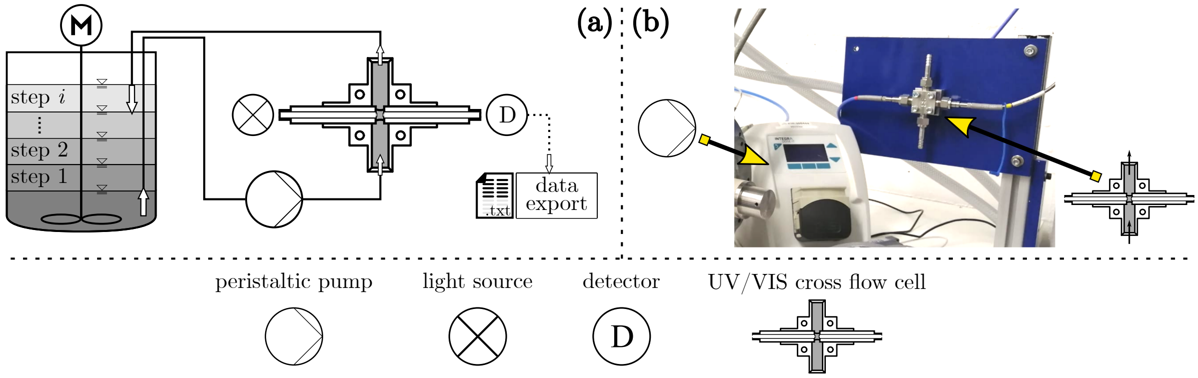

Figure S1. Schematic view of the calibration setup (a). Illustration of the maunted UV/vis cross flow cell and the peristaltic pump used to acquire a calibration dataset (b). Components are also depicted and named in the bottom legend.

The sensor acquired and saved extinction data continuously and therefore overlooked any change in extinction over time. During dilution, a pre-calculated volume of demineralized water was added to the beaker in multiple steps. This resulted in several batches with a defined solids volume fraction of PMMA NPs. All set particle concentrations are listed in Tab.(S1). Prior to each increment, care was taken to ensure that a stationary spectrum was acquired. The reason for this was that the calibration data was extracted at the respective plateaus of the dilution steps. This operation is visualized in Fig.(S2) in the top left. Recorded spectra are plotted as a three-dimensional plane over time. The decrease in extinction is connected to the added demineralized water into the stirred suspension. Black and gray lines indicate the mentioned plateaus of constant extinction. Later at these exact positions in a time window of 3.6 s, extinction data was extracted to calculate a mean extinction spectrum.

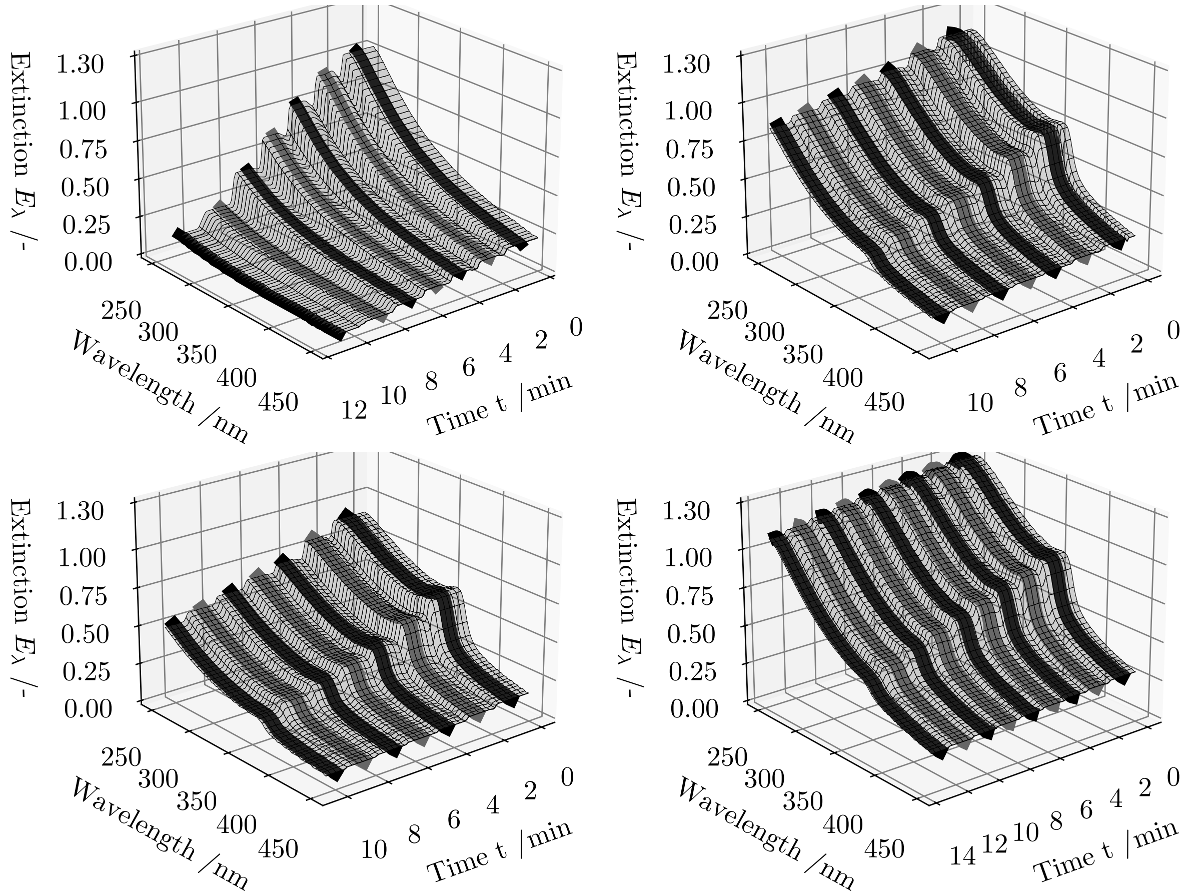

Figure S2. Spectra acquired from four continuous dilution series. (a) PMMA suspension diluted with demineralized water (caC). Zinc oxide (ZnO) suspension diluted with PMMA suspension with solids volume fraction of (b) $\phi_{\text{PMMA}} = 4.7 \cdot 10^{-4}$, (c) $\phi_{\text{PMMA}} = 9.4 \cdot 10^{-4}$ and (d) $\phi_{\text{PMMA}} = 1.411 \cdot 10^{-4}$ (calF-1 to calF-3). Solid lines mark plateaus with constant material compositions where multiple spectra are isolated, averaged and saved in the corresponding calibration dataset. Black lines refer to isolated samples used as training data in soft sensor model diagnostic. Grey lines highlight the extinction data used in LOO cross validation.

This data was then labeled with the corresponding solids volume fraction and added to the calibration dataset *caC*. The lines are colored according to the use of spectral information in the diagnostic procedure described in the supported publication. Black lines are spectra that function as training data and gray lines are used for model validation.

1.2 Recording of dilution series *caF*

The experimental setup described in section 1.1 also applies to calibration data acquisition for multidimensional fractionation of mixed suspensions containing both PMMA and ZnO nanoparticles. The whole calibration data set contained 23 averaged extinction spectra. Each curve again corresponded to a measured signal plateau at a pre-calculated relative solids volume fraction of both suspended materials. Concentration data of these different material composition instances is specified in S1. The procedure involved three separate dilutions. First, a mixture with $\phi_{\mathrm{ZnO}} = 5.0 \cdot 10^{-5}$ and three separate concentrations of polymer NPs ($\phi_{\mathrm{PMMA,caF-1}} = 4.75 \cdot 10^{-4}$, $\phi_{\mathrm{PMMA,caF-2}} = 9.4 \cdot 10^{-4}$, $\phi_{\mathrm{PMMA,caF-3}} = 1.4 \cdot 10^{-3}$) was constantly stirred in the beaker. During dilution, each individual batch was diluted with a PMMA suspension with the same solids volume fraction. This ensured that only the particle concentration in regard to ZnO was lowered.

Table S1. Solids volume fractions of each sample extracted from the continuous dilution series displayed in Fig.(S2)

		solids volume fraction ϕ /- $\phi_{\mathrm{PMMA}} \mid \phi_{\mathrm{ZnO}}$			
		calC	caF-1	caF-2	caF-3
Sample number	1	$1.0 \cdot 10^{-3}$ — 0.0	$4.7 \cdot 10^{-4}$ — $5.0 \cdot 10^{-5}$	$9.4 \cdot 10^{-4}$ — $5.0 \cdot 10^{-5}$	$1.4 \cdot 10^{-3}$ — $5.0 \cdot 10^{-5}$
	2	$8.0 \cdot 10^{-4}$ — 0.0	$4.7 \cdot 10^{-4}$ — $4.0 \cdot 10^{-5}$	$9.4 \cdot 10^{-4}$ — $4.0 \cdot 10^{-5}$	$1.4 \cdot 10^{-3}$ — $4.5 \cdot 10^{-5}$
	3	$6.0 \cdot 10^{-4}$ — 0.0	$4.7 \cdot 10^{-4}$ — $3.0 \cdot 10^{-5}$	$9.4 \cdot 10^{-4}$ — $3.0 \cdot 10^{-5}$	$1.4 \cdot 10^{-3}$ — $4.0 \cdot 10^{-5}$
	4	$4.0 \cdot 10^{-4}$ — 0.0	$4.7 \cdot 10^{-4}$ — $2.5 \cdot 10^{-5}$	$9.4 \cdot 10^{-4}$ — $2.5 \cdot 10^{-5}$	$1.4 \cdot 10^{-3}$ — $3.5 \cdot 10^{-5}$
	5	$3.0 \cdot 10^{-4}$ — 0.0	$4.7 \cdot 10^{-4}$ — $2.0 \cdot 10^{-5}$	$9.4 \cdot 10^{-4}$ — $2.0 \cdot 10^{-5}$	$1.4 \cdot 10^{-3}$ — $3.0 \cdot 10^{-5}$
	6	$2.0 \cdot 10^{-4}$ — 0.0	$4.7 \cdot 10^{-4}$ — $1.5 \cdot 10^{-5}$	$9.4 \cdot 10^{-4}$ — $1.5 \cdot 10^{-5}$	$1.4 \cdot 10^{-3}$ — $2.5 \cdot 10^{-5}$
	7	$1.0 \cdot 10^{-4}$ — 0.0	$4.7 \cdot 10^{-4}$ — $1.0 \cdot 10^{-5}$	$9.4 \cdot 10^{-4}$ — $1.0 \cdot 10^{-5}$	$1.4 \cdot 10^{-3}$ — $2.0 \cdot 10^{-5}$
	8	-	-	-	$1.4 \cdot 10^{-3}$ — $1.5 \cdot 10^{-5}$
	9	-	-	-	$1.4 \cdot 10^{-3}$ — $1.0 \cdot 10^{-5}$

Data extraction at the individual plateaus of constant extinction is the same as with calibration dataset *caC*. For diagnostic purposes, the merged extinction data, labeled with material composition information, was split up for model diagnostics by cross validation of the regression model.

2 FURTHER DETAILS ON THE PARTICLE SYSTEMS

2.1 Particle morphology and qualitative assessment of fractionation outcome

In addition to a quantitative analysis of all samples for their relative solids volume fraction and material composition, three additional dispersions were analyzed qualitatively by scanning electron microscopy (SEM). The strongly diluted suspensions were applied to a membrane and the continuous phase was removed by filtration and drying. Image analysis of these specimens resulted in the exposures shown in Fig.(S3). On the left hand side (Fig.(S3a) the feed of fractionation experiment *expF* is shown. To highlight the different materials, the spherical PMMA particles were colored blue and some ZnO aggregates were colored green. The second image (Fig.(S3b) originated from the analysis of an overflow sample taken after 30 min during *expF* (C = 50000). Regarding this sample, it was challenging to find remaining ZnO NPs since they were separated effectively during centrifugation. What remained and is visible in the image are a exemplary collection of the fine fraction of PMMA. The last SEM image (Fig.(S3c) of this collection depicts the coarse fraction of *expF* harvested in the front part of the rotor near the inlet. A small amount of sediment was re-dispersed in demineralized water and prepared for the SEM analysis. In the membrane section shown, significantly larger aggregates and agglomerates of ZnO can be seen. Also identifiable are some of the coarser polymer particles that have been separated.

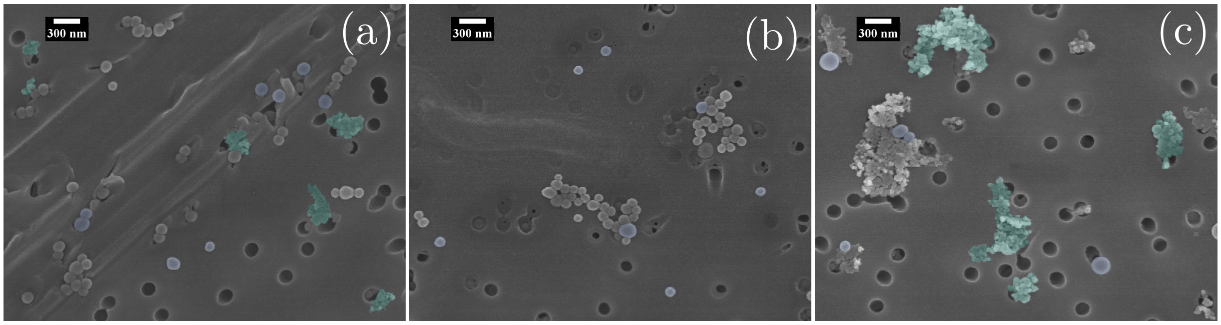

Figure S3. Qualitative SEM image analysis of particle suspensions extracted from experiment *expF*: (**a**) Mixture of PMMA and ZnO nanoparticles (Feed). (**b**) Overflow sample after processing in tubular centrifuge $C = 50000$. (**c**) Sample prepared with harvested sediment in centrifuge rotor.

The analysis qualitatively confirms the findings of the publication that the denser and coarser material ZnO can be separated much more efficiently during centrifugation in the tubular centrifuge.

2.2 Suspension stability

In order to investigate the classification and fractionation according to size and material properties, it was essential to ensure a constant particle size distribution during the experimental trials. This implied that both pure substance systems as well as their mixtures had to be sufficiently stabilized to inhibit homo- or hetero agglomeration. This was achieved by the addition of 0.1 mM sodium hexametaphosphate ($Na_6P_6O_{18}$). To investigate the suspension stability in a worst-case estimation, a mixture with the initial feed concentrations of experiment *expF* was prepared. The mixed dispersion was continuously stirred and cycled through the UV/vis cross flow cell for more than 5 hours and the respective extinction spectrum was monitored constantly. Five of the recorded spectra at ascending point in time are compared in Fig.(S4).

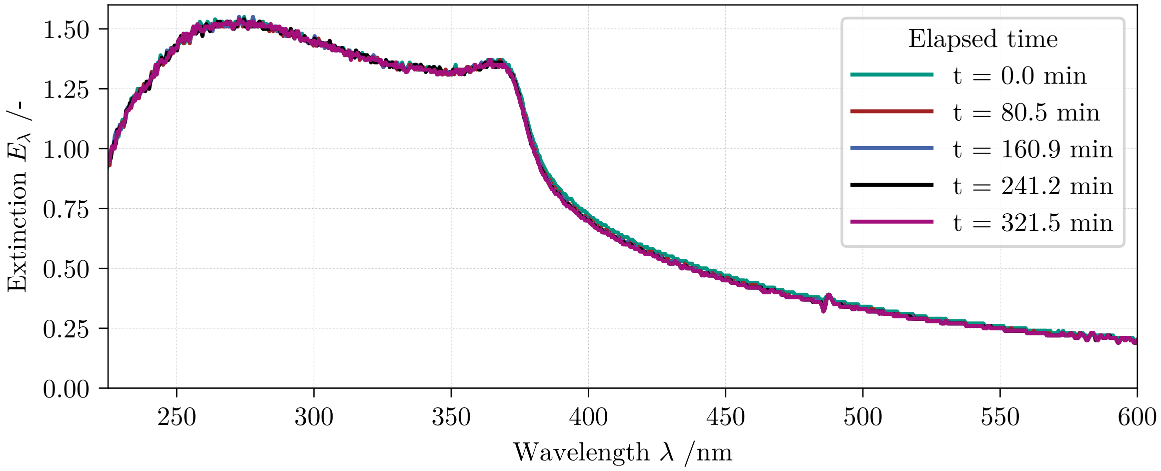

Figure S4. Five extinction spectra of the mixed feed suspension used in continuous fractionation monitoring. Data acquisition was performed in the experimental setup shown in Fig.(S1). The dispersion was constantly stirred with a magnetic stirrer for more than five hours. Each curve is labeled with the elapsed time of the test measurement.

The plotted curves are congruent which indicates that no agglomeration of primary particles occurs. All experimental studies regarding fractionation were performed in this observed time period. Consequently it can be assumed that the separation outcome during fractionation (*expF*) and classification (*expC*) is not affected by particle agglomeration and that the suspension is sufficiently stabilized by the amount of $Na_6P_6O_{18}$ dissolved in the suspensions.

3 FURTHER DETAILS ON QUANTITATIVE ANALYSIS OF SOLIDS VOLUME FRACTION AND SUSPENSION COMPOSITION

3.1 ICP-OES data and quantitative suspension composition calculation

As stated in the main text, an inductively coupled plasma optical emission spectrometry (ICP-OES) analysis was carried out to evaluate the relative solids volume fraction of both suspended materials in the prepared feed and in the overflow (*expF*). Since only the pure content of zinc (Zn) was evaluated by the ICP-OES analysis, the concentration of the other constituents was calculated with the help of the performed measurement of the total amount of suspended solids (TAS). The closing condition of mass balance and the stochiometric ratio between zinc and oxygen lead to the results listed in both Tab.(S2) and Tab.(S3). Every row entry is based on a threefold measurement. Individual runs of the two-fold fractionation experiment *expF* are highlighted.

Table S2. Concentration data for pure Zinc (Zn) measured by the ICP-OES analysis for the feed sample and two overflow samples of experiment *expF*. Zinc oxide (ZnO) content was determined using the stoichiometric ratio and the molar mass of both zinc and oxygen.

		Zinc (Zn^{2+})	Oxygen (O^{2-})	Zinc oxide (ZnO)		
Centrifugal number	Run	Concentration /mgml^{-1}	Concentration /mgml^{-1}	Concentration /mgml^{-1}	Solids volume fraction /-	Product loss P /-
C=0 (feed)		326.6 ± 5.8	79.170 ± 1.418	402.800 ± 2.313	$7.180 \cdot 10^{-5} \pm 4.124 \cdot 10^{-7}$	
C=10000 (weir)	1	60.9 ± 1.3	14.895 ± 0.319	75.784 ± 1.389	$1.351 \cdot 10^{-5} \pm 2.477 \cdot 10^{-7}$	$0.188 \pm 7.40 \cdot 10^{-3}$
	2	61,3 ± 1.2	14.984 ± 0.289	76.236 ± 1.253	$1.359 \cdot 10^{-5} \pm 2.234 \cdot 10^{-7}$	$0.189 \pm 7.40 \cdot 10^{-3}$
C=30000 (weir)	1	19.1 ± 0.5	4.668 ± 0.115	23.749 ± 1.342	$4.233 \cdot 10^{-6} \pm 2.392 \cdot 10^{-7}$	$0.059 \pm 2.50 \cdot 10^{-3}$
	2	19.4 ± 0.5	4.746 0.120	24.146 ± 1.384	$4.304 \cdot 10^{-6} \pm 2.467 \cdot 10^{-7}$	$0.060 \pm 2.59 \cdot 10^{-3}$
C=50000 (weir)	1	12.1 ± 0.4	2.959 ± 0.100	15.055 ± 1.784	$2.684 \cdot 10^{-6} \pm 3.181 \cdot 10^{-7}$	$0.037 \pm 1.93 \cdot 10^{-3}$
	2	12.0 ± 0.3	2.947 ± 0.072	14.996 ± 1.300	$2.673 \cdot 10^{-6} \pm 2\text{-}318 \cdot 10^{-7}$	$0.037 \pm 1.58 \cdot 10^{-3}$

Table S3. Concentration data related to the TAS of a sample determined by gravimetric measurements. Calculated solids volume fraction of PMMA was determined with respective values listed in Tab.(S2) and the closing condition of mass balance in each sample.

		Total amount of solids (TAS)		Polymethylmethacrylate (PMMA)		
Centrifugal number	Run	Concentration /mgml^{-1}	Product loss /-	Concentration /mgml^{-1}	Solids volume fraction /-	Product loss /-
C=0 (feed)		2348.8 ± 27.081		1946.00 ± 29.40	$1.631 \cdot 10^{-3} \pm 2.4639 \cdot 10^{-5}$	
C=10000 (weir)	1	1626.7 ± 11.542	0.693 ± 0.013	1550.92 ± 12.93	$1.300 \cdot 10^{-3} \pm 1.0840 \cdot 10^{-5}$	0.797 ± 0.019
	2	1610.9 ± 9.698	0.686 ± 0.012	1534.66 ± 10.95	$1.286 \cdot 10^{-3} \pm 9.1795 \cdot 10^{-6}$	0.789 ± 0.015
C=30000 (weir)	1	1156.0 ± 3.872	0.492 ± 0.007	1132.25 ± 5.214	$9.491 \cdot 10^{-4} \pm 4.3705 \cdot 10^{-6}$	0.582 ± 0.009
	2	1190.9 ± 13.895	0.507 ± 0.012	1166.75 ± 15.28	$9.780 \cdot 10^{-4} \pm 1.2807 \cdot 10^{-5}$	0.600 ± 0.018
C=50000 (weir)	1	868.78 ± 3.865	0.370 ± 0.006	853.73 ± 5.649	$7.156 \cdot 10^{-4} \pm 4.7355 \cdot 10^{-6}$	0.439 ± 0.014
	2	884.260 ± 7.686	0.376 ± 0.008	869.26 ± 8.986	$7.286 \cdot 10^{-4} \pm 7.5325 \cdot 10^{-6}$	0.447 ± 0.017

3.2 Continuous monitoring of fine fraction composition: Evaluation with inferior model inputs

The main findings of the presented paper state that the prediction error of the soft sensor is reduced by an adequate selection of model inputs. In light of this, three wavelength ranges were introduced at which the multiple linear regression model (MLR) was constructed. It was highlighted, that feature range FR1 and FR3 were inferior to FR2 since the prediction error regarding the solids volume fraction is greater. This decrease in predictive strength is visualized by Fig.(S5) and Fig.(S6) respectively.

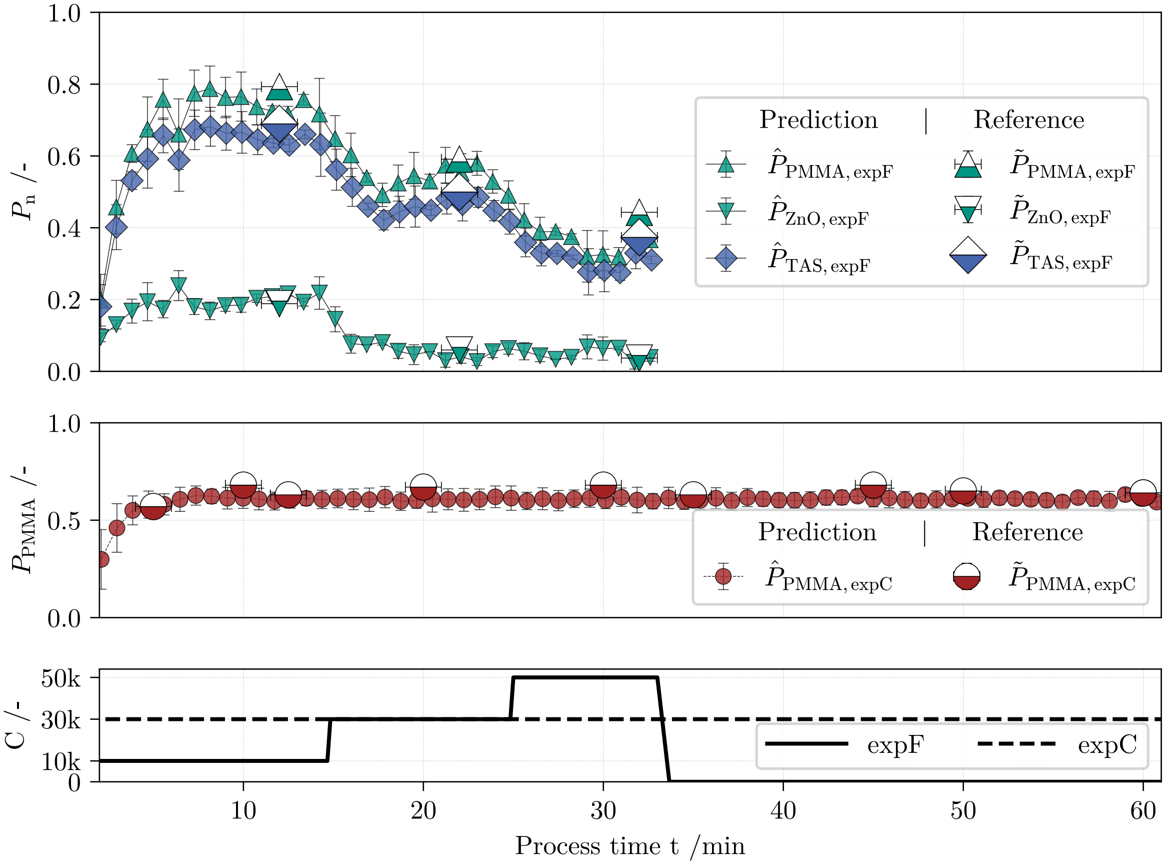

Figure S5. Comparison of sensor output (solid symbols) and laboratory analysis (half-filled symbols) of the product loss in fractionation (a) and classification (b) monitoring over the elapsed process time. The set centrifugation number is drawn on shared abscissa (c). The soft sensor output is based on the corresponding calibration data set of feature range FR1.

By reducing the model input as in FR1, the algorithm can qualitatively predict the expected product loss during classification and fractionation. However, the output signal becomes less precise, which is clearly shown in the case of the more difficult monitoring fractionation. In regard FR3, it was stated that there is no distinguishable signals information identifying ZnO in these band of the UV/vis spectrum. Consequently, the constructed linear model performs poorly in online fractionation monitoring. In case of classification monitoring, however, the build calibration dataset was sufficient for an moderate prediction of the solids volume fraction of PMMA.

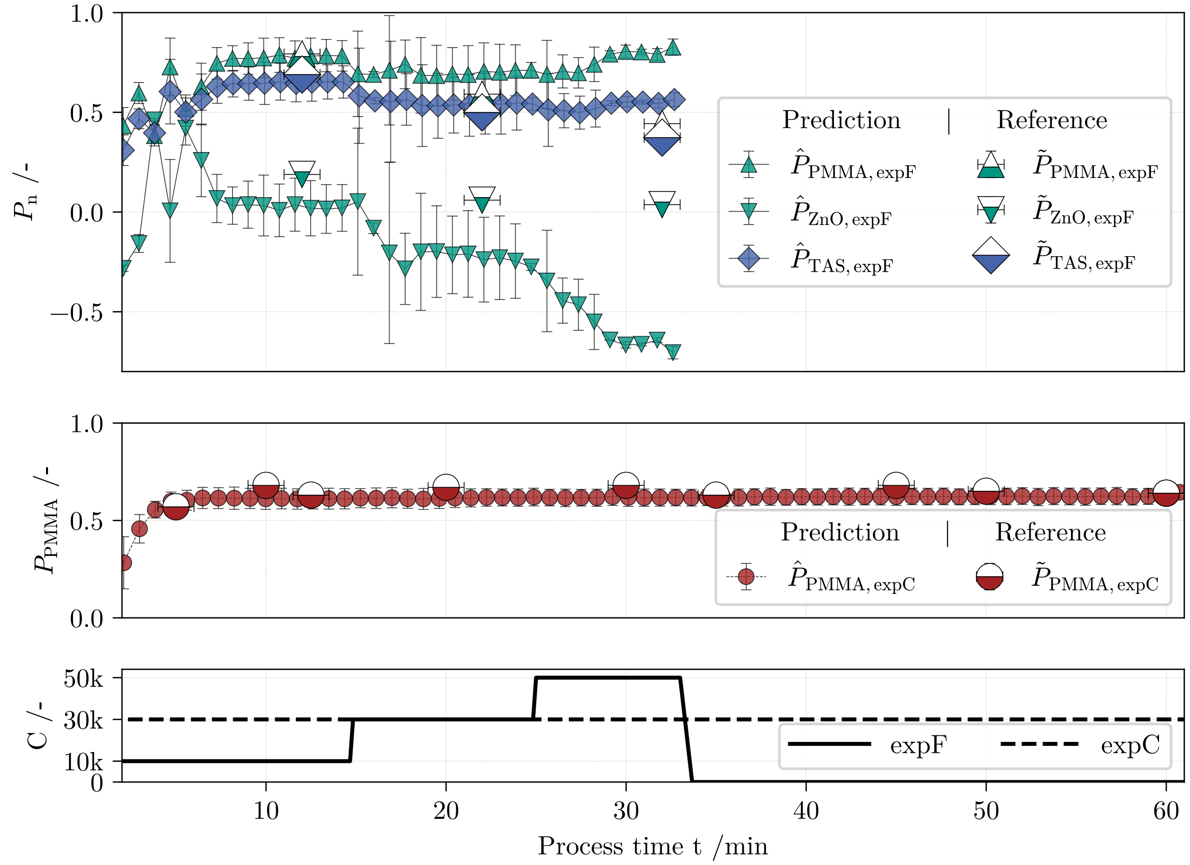

Figure S6. Comparison of sensor output (solid symbols) and laboratory analysis (half-filled symbols) of the product loss in fractionation (a) and classification (b) monitoring over the elapsed process time. The set centrifugation number is drawn on shared abscissa (c). The soft sensor output is based on the corresponding calibration data set of feature range FR3.
